# Guided electrocatalyst design through in-situ techniques and data mining approaches

**DOI:** 10.1186/s40580-025-00484-3

**Published:** 2025-04-18

**Authors:** Mingyu Ma, Yuqing Wang, Yanting Liu, Shasha Guo, Zheng Liu

**Affiliations:** 1 School of Materials Science and Engineering, Nanyang Technological University, Singapore, 639798 Singapore; 2 School of Chemistry, Chemical Engineering and Biotechnology, Nanyang Technological University, Singapore, 637616 Singapore; 3 Department of Materials Science and Engineering, National Tsing Hua University, Hsinchu, 30013 Taiwan; 4 Department of Chemistry and Chemical Biology, Cornell University, Ithaca, NY 14853-1301 USA; 5 CINTRA CNRS/NTU/THALES, UMI 3288, Research Techno Plaza, Singapore, 639798 Singapore; 6 Institute for Functional Intelligent Materials, National University of Singapore, Singapore, 117544 Singapore

**Keywords:** In-situ experimental techniques, Data mining, Catalytic mechanism, Mechanism guidance, Structural-property relationship

## Abstract

Intuitive design strategies, primarily based on literature research and trial-and-error efforts, have significantly contributed to advancements in the electrocatalyst field. However, the inherently time-consuming and inconsistent nature of these methods presents substantial challenges in accelerating the discovery of high-performance electrocatalysts. To this end, guided design approaches, including in-situ experimental techniques and data mining, have emerged as powerful catalyst design and optimization tools. The former offers valuable insights into the reaction mechanisms, while the latter identifies patterns within large catalyst databases. In this review, we first present the examples using in-situ experimental techniques, emphasizing a detailed analysis of their strengths and limitations. Then, we explore advancements in data-mining-driven catalyst development, highlighting how data-driven approaches complement experimental methods to accelerate the discovery and optimization of high-performance catalysts. Finally, we discuss the current challenges and possible solutions for guided catalyst design. This review aims to provide a comprehensive understanding of current methodologies and inspire future innovations in electrocatalytic research.

## Graphical abstract

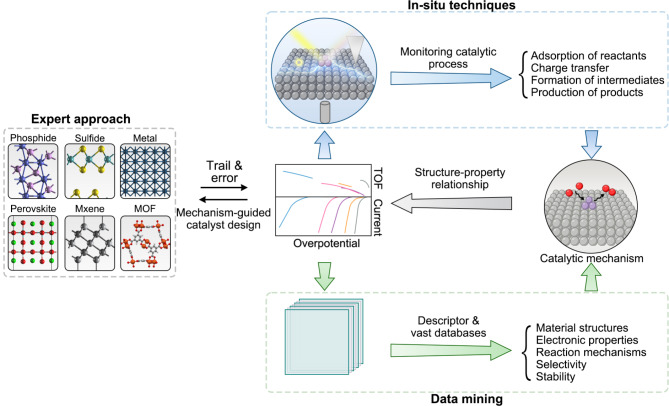

## Introduction

The growing world population and accelerating industrialization have driven increased energy demand, primarily reliant on limited fossil fuels, ultimately leading to resource depletion [1]. Additionally, this demand has escalated greenhouse gas emissions, climate change, and extreme weather events that threaten human livelihoods [2]. These pressures have also resulted in an unprecedented decline in biodiversity, occurring 1,000 times faster than any other period in recorded history [3]. In this context, clean and affordable electrocatalytic technologies have emerged as viable alternatives for transitioning the global economy away from fossil fuels [4]. For instance, while green hydrogen produced via water electrolysis is currently more expensive than conventional fossil fuels, this situation can be reversed by carefully designing crucial technology components—specifically, the electrocatalysts [5, 6].

To date, most advancements in the field of electrocatalysis have relied on experimental material science based on intuitive design strategies, without clear guidance. Typically, for a specific reaction, a material scientist begins with conducting thorough literature research to establish a structure-property relationship from existing studies mentally. The scientist then conceptualizes a new catalyst structure, designs appropriate synthesis strategies, and subsequently tests the performance of the materials for the intended reaction. This traditional approach is time-consuming and inconsistent and relies heavily on individual expertise.

As a result, the need for design guidance in catalyst development has become increasingly evident. Such an approach began to emerge following the introduction of the “active site” concept by Taylor in 1932 [7], which highlighted that only a small fraction of surface sites are responsible for catalytic activity. This insight redirected research efforts towards identifying the specific nature and behavior of these active sites, further establishing precise structure-property relationships. The development of advanced operando/in-situ techniques, such as X-ray absorption spectroscopy (XAS) [8, 9], Raman spectroscopy, transmission electron microscopy (TEM), and other methods, has provided revolutionary tools for gaining a detailed understanding of electrocatalysis at the nanometer or even atomic level, potentially guiding rational electrocatalysts design. For example, Granozzi et al. explored the catalytic activity of single Fe atoms through operando electrochemical scanning tunneling microscopy (EC-STM) with atomic-scale spatial resolution, revealing variations in the activity of Fe atoms under different configurations [10].

Alternatively, data mining offers a robust method for constructing structure-property relationships by leveraging descriptors and vast databases. The electrocatalysis field has accumulated a significant amount of domain knowledge, with over 835,000 publications since 1900 covering material structures, electronic properties, reaction mechanisms, selectivity, stability, and more. Over the past two decades, continuous validation against empirical observations by various research groups has transformed fragmented insights into comprehensive theoretical foundations, such as the volcano plot and electronic structure theories, which are now central to understanding catalytic activities. These foundational insights can serve as valuable descriptors in data-driven approaches. Thanks to increased computational power and the development of large language models, we have entered an era where data mining can systematically analyze and extract meaningful patterns from this wealth of information, significantly enhancing catalyst design and discovery.

In light of these advancements, the subsequent sections of this review will explore guided electrocatalyst design for low-dimensional catalysts through operando/in-situ techniques and data mining methods. We will discuss how operando methods can be employed to monitor the catalytic processes, providing unknown insights and guidance for catalyst optimization. Additionally, we will examine how data mining utilizes extensive datasets and domain knowledge to predict catalytic performance and discover new materials. Last, we will present personal perspectives on the challenges and opportunities of these two approaches in electrocatalyst design, highlighting their potential to accelerate the transition to sustainable energy solutions.

## In-situ techniques for unveiling catalytic processes

In-situ techniques have become indispensable for probing surface catalytic processes on low-dimensional catalysts. By monitoring in real-time, these approaches allow researchers to observe key steps such as reactant adsorption, charge transfer, and the formation of intermediates and products, which are not yet fully understood. Furthermore, they play a pivotal role in establishing the structure-property relationships by correlating the physical and electronic structures of catalysts with obtained spatial heterogeneity of monitored signals. This direct evidence deepens the fundamental understanding of catalytic processes and informs the design of more effective catalysts. For example, operando studies have been instrumental in revealing the stepwise mechanisms of CO_2_ reduction (CO_2_RR) and hydrogen evolution reactions (HER) [11–13], enabling the design of catalysts with optimized active sites tailored for these specific reactions.

In the following sections, we will first classify the emergence of in-situ techniques and then demonstrate their capability to explore critical aspects in surface catalysis, including the identification of active sites, reaction mechanisms and pathways, and product formation.

### Classification of in-situ techniques by detected signals

Numerous in-situ experimental techniques have been developed to complementally “visualize” catalytic reactions across both time and spatial dimensions. These techniques can be categorized into five groups based on the types of detected signals: photonic techniques, electronic methods, electrochemical current measurements, fluorescence-based methods, and mass spectrometry techniques.

(1) Photonic techniques include XAS [14], X-ray photoelectron spectroscopy (XPS) [15], Raman spectroscopy [16, 17], infrared (IR) spectroscopy [18], grazing incidence X-ray diffraction (GIXRD) [19], and ultraviolet-visible (UV-Vis) spectroscopy [20]. These techniques detect surface information of catalysts under working conditions by collecting optical signals.

(2) Electronic methods encompass transmission electron microscopy [21], scanning electron microscope (SEM) [22], electron energy loss spectrum (EELS) [23, 24], atomic electron tomography [25], and EC-STM [10, 26]. These methods gather surface or bulk information of catalyst during catalytic reactions via measuring changes in electronic signals.

(3) Electrochemical current mapping includes scanning electrochemical microscopy (SECM) [27] and scanning electrochemical cell microscopy (SECCM) [28]. By scanning material surfaces and recording electrochemical currents simultaneously, these techniques can directly visualize the spatial distribution of heterogeneity in terms of reactivity.

(4) Fluorescence super-resolution microscopy is capable of visualizing the distribution of catalytic activity with a nanometer resolution. Several methods have been reported to achieve this, including redox fluorogenic reactions for imaging reduction and oxidation activity [29, 30], competitive adsorption for visualizing reactivity in non-fluorogenic reactions [31], and nanobubble mapping to describe activity distribution in gas-evolving reactions [32]. These approaches enable detailed mapping of catalytic processes and provide insights into the spatial activity patterns of catalysts.

(5) Mass spectrometry techniques include on-line differential electrochemical mass spectrometry (DEMS) [33] and on-line inductively coupled plasma mass spectrometry (ICP-MS) [34]. These techniques monitor the catalytic reactants, intermediates, and products by analyzing peak positions, peak intensities, and temporal variations in mass spectra.

### Identification of active sites

Active sites in heterogeneous catalysis are extraordinarily complex, as they are influenced by a multitude of factors, including chemical composition, electronic structure, local atomic arrangement, as well as interaction with reactants and electrolyte environment [35]. This complexity makes it challenging to fully understand and optimize their behaviors for specific reactions. Consequently, the identification and characterization of active sites are paramount for effectively engineering them—whether by increasing their population or enhancing their intrinsic activity. To achieve this, various in-situ techniques are developed that can probe active sites at multiple scales, from nanometric to atomic resolutions and from picoseconds to seconds [36]. These methods provide valuable insights into their structure and functionality, which will be discussed as follows.

**Fig. 1:**
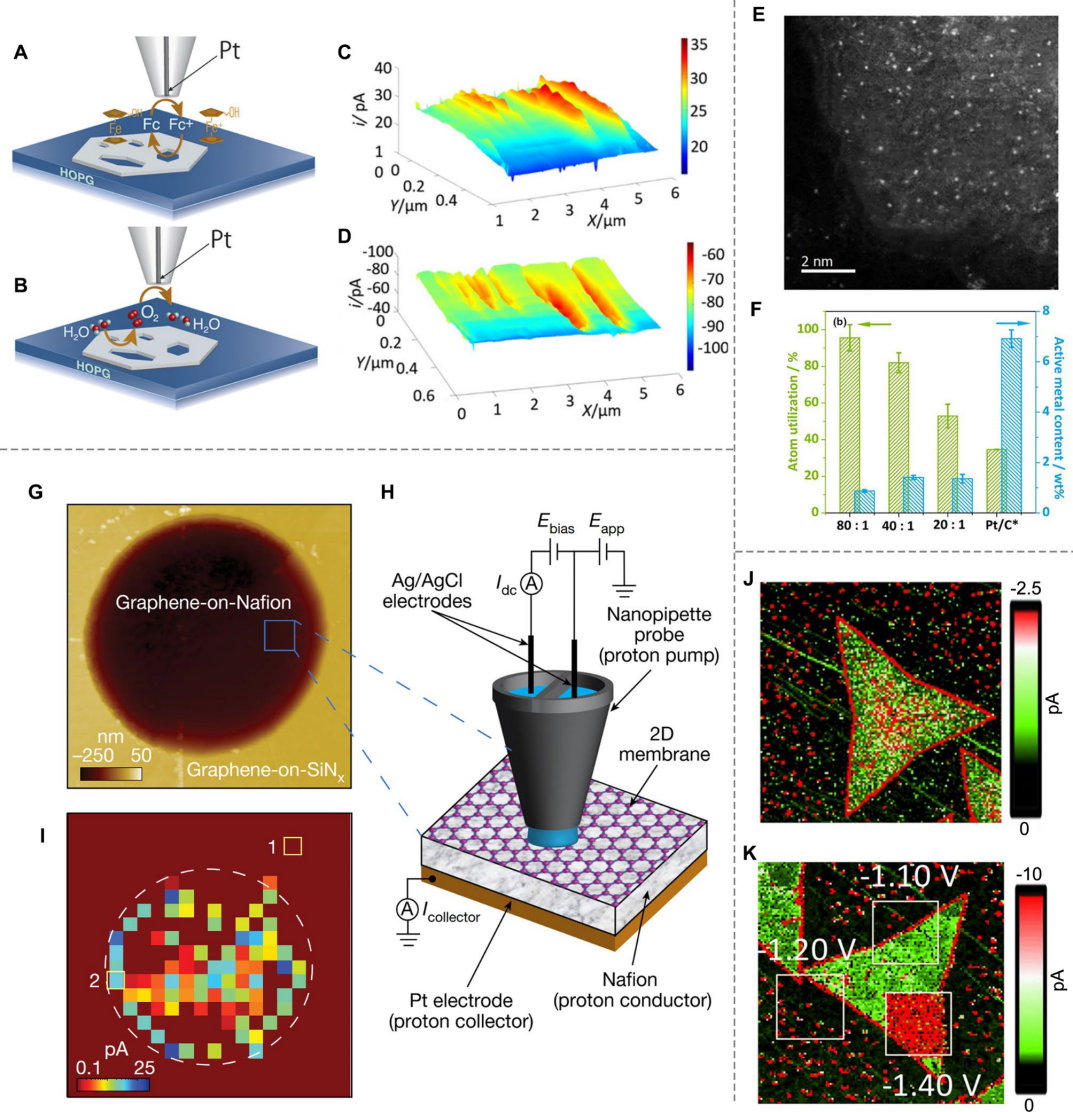
Atomic-scale identification of active sites with SECM and SECCM techniques. (**A**-**B**) Schematic representation of (**A**) positive feedback produced by oxidation/reduction of ferrocene methanol (Fc) and (**B**) substrate generation/tip collection of dioxygen at NiO nanosheet. (**C**-**D**) SECM imaging of NiO nanosheet with the HOPG as the substrate at the (**C**) Feedback mode and (**D**) SG/TC mode. Reproduced with permission from [37]. Copyright 2019, National Academy of Sciences. (**E**) High-angle annular dark-field scanning transmission electron microscopy (HAADF STEM) image of PPy-CuPcTs (40:1). (**F**) Atom utilization and active metal content based on SI-SECM titration and ICP-MS results. Reproduced with permission from [39]. Copyright 2020, Elsevie. (**G**) Atomic force microscopy (AFM) image of the designed setup of a 2-μm-diameter circular aperture in the SiN_x_ substrate covered with monolayer graphene. (**H**) Schematic of SECCM setup. (**I**) Example of SECCM maps (I_collector_ maps) for apertures covered with graphene. Reproduced with permission from [28]. Copyright 2023, Springer Nature. (**J**-**K**) SECCM current maps of (**J**) 2D MoS_2_ and (**K**) 2D MoS_2_ after electrochemical activation on HOPG substrate. Reproduced with permission from [46]. Copyright 2020, Wiley

For example, SECM can pinpoint active sites on the surface of catalysts by scanning a miniaturized electrode tip very close to the catalyst surface with a nanometric resolution. In particular, Richards and co-workers directly mapped the oxygen evolution reaction (OER) activity catalyzed by a semi-two-dimensional (2D) NiO catalyst using the operando SECM technique with sub-20 nm spatial resolution [37]. As shown in Fig. 1A, the reduction of Fc^+^ to Fc (feedback mode) was employed to map the defective areas of 2D NiO, attributed to the faster mediator regeneration rate at the exposed highly oriented pyrolytic graphite (HOPG) substrate. Impressively, the substrate generation/tip collection (SG/TC) image of the same area revealed higher OER reactivity at the 2D NiO surface compared to the substrate area, as demonstrated by mapping the catalytic current (Fig. 1B). Furthermore, they elucidated that the OER catalytic activity at the edge of NiO was significantly higher than that at fully coordinated surfaces (Fig. 1C-D).

In another case study, SECM was employed to examine the atom-utilization efficiency of single-atom catalysts (SAC), which is predicted to be 100% but lacks direct experimental evidence [38]. Xiao et al. utilized the operando surface-interrogation SECM (SI-SECM) technique to access the atom utilization of Cu single atoms in copper phthalocyanine-3, 4’, 4’’, 4’’’-tetrasulfonic acid tetrasodium salt doped polypyrrole film (PPy-CuPcTs) SAC during the oxygen reduction reaction (ORR) process (Fig. 1E) [39]. They reported that the atom utilization of Cu sites could reach up to 95.6%, substantially higher than that of commercial Pt/C catalysts (34.6%), as shown in Fig. 1F.

While significant advancements in active site detection have been achieved using SECM, it does have several underlying limitations: (i) Limited temporal resolution is caused by the low speed of the scanning probe, a necessary feature to minimize scanning-induced convection, which would otherwise disrupt the diffusion of reactants and products [27]. (ii) Restricted research subjects, as a flat surface geometry is essential to protect the nanometer-sized tip.

To address these limitations, Unwin and colleagues initially developed the SECCM technique to monitor the electrochemical reactions at the nanoscale [39, 40]. By using a nanopipette probe to confine the electrolyte, this technique forms a highly localized electrochemical cell [41–44], enabling finer control over reaction area, higher spatial resolution down to ∼ 20 nm, higher temporal resolution down to ∼ 3 milliseconds [45], and greater flexibility in surface geometries, especially those with higher roughness [28, 41, 46]. For example, Matsue et al. exploited SECCM to investigate the difference in HER activity across edge, terrace, and heterojunction of 2D MoS_2_ and WS_2_ single flakes [46]. As shown in Fig. 1J, their results revealed that the HER activity of MoS_2_ monolayer sheets is strongly influenced by structural morphology. Notably, the edge regions exhibit enhanced performance over the basal plane regions, achieving a lower overpotential (0.94 V versus reversible hydrogen electrode, denoted as V_RHE_, compared to 1.06 V_RHE_) and a more favorable Tafel slope (130 mV dec⁻¹ compared to 150 mV dec⁻¹) (Fig. 1K).

It is worth noting that SECCM has the potential to address ongoing debates in the field of confined catalysis. Confined catalysis, which refers to reactions occurring in nanoconfined reactors, has demonstrated intriguing capabilities for modulating surface reactions [47–49]. For instance, in the context of HER, researchers have debated whether electron tunneling occurs from the catalyst to the 2D confined layer or whether protons penetrate through the 2D layer to reach the catalyst surface [50, 51]. SECCM provides a promising technique for investigating this issue further. As an early attempt, Unwin’s group constructed a Pt-Nafion-2D crystals (such as graphene and h-BN) sandwich structure and studied proton transport through 2D layers by real-time monitoring of the HER current using SECCM (Fig. 1G-H) [28]. Their findings demonstrate that nanoscale morphology and the associated strain and curvature play a crucial role in proton transport through typical two-dimensional crystals (Fig. 1I).

In addition to detection through electrochemical current signals, fluorescence-based techniques offer an alternative method for active site detection at the nanometer scale. Single-molecule fluorescence (SMF) imaging, which offers nanometric spatial resolution (typically 10–20 nm) and millisecond temporal resolution, has been employed to study catalytic heterogeneity [30, 31, 52–56].

**Fig. 2:**
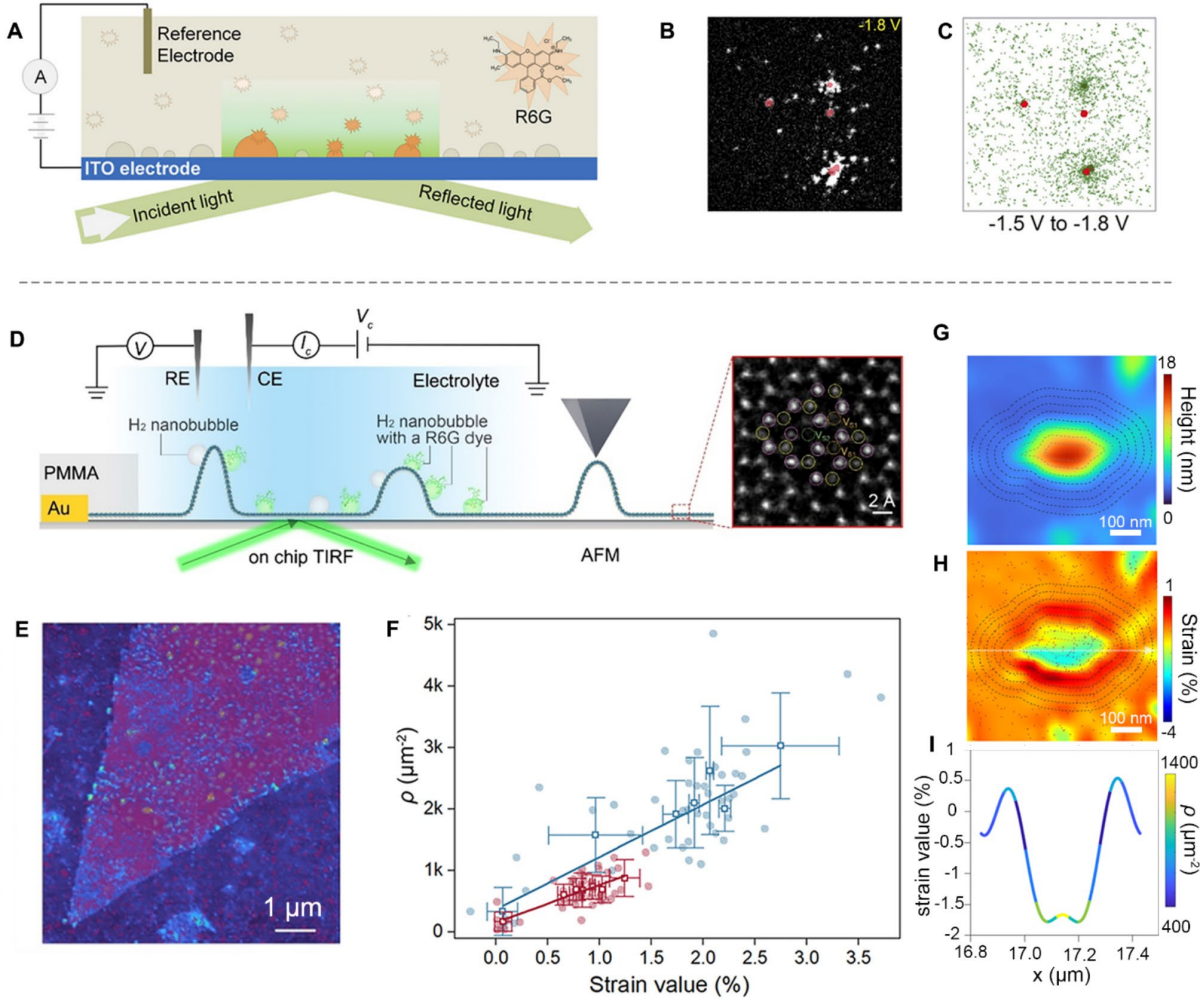
Identification of active sites with the SMF imaging. (**A**) Schematic representation of the experimental setup used for imaging H_2_ nanobubbles during electrocatalytic water splitting. (**B**) TIRF image at the applied voltage of -1.8 V. (**C**) Scatter plots depicting the accumulated spatial distribution of H_2_ nanobubbles observed during the potential scan from − 1.5 V to − 1.8 V. Reproduced with permission from [32]. Copyright 2018, National Academy of Sciences. (**D**) Schematic diagram of the on-chip TIRF setup. (**E**) Overlay of local activity and AFM topology of monolayer MoS_2_ with a high density of protrusions. (**F**) The plot of local activity represented by nanobubble density as the function of tensile (blue) and compressive (red) strain. (**G**) AFM topology of a typical single protrusion. Note that the dotted polygonal rings denote the boundaries of radial segments (width: 39 nm). (**H**) Overlay image of strain map and nanobubbles (purple). (**I**) Averaged nanobubble density versus strain value across the white dotted line in panel H. Reproduced with permission from [58]. Copyright 2024, American Chemical Society

For example, utilizing the transient adsorptive behavior of Rhodamine 6G (R6G), which is a highly fluorescent rhodamine family dye, at the gas-liquid interface, the SMF imaging technique enables visualization of the nucleation and growth of nanobubbles at the electrode/electrolyte interface. This capability makes it a powerful tool for identifying active sites in gas-evolving electrocatalytic reactions. For instance, Zhang et al. studied the active site distribution on indium tin oxide (ITO) supported metal nanoparticles by visualizing R6G as indicators for H_2_ nanobubbles under a total internal reflection configuration (TIR) (Fig. 2A, C) [32]. Their results show that hydrogen nanobubbles were generated at very early stages (i.e., > 500 mV before reaching the thermodynamic redox potential of HER) on the ITO electrode (Fig. 2B). Later on, they employed an Au/Pd metal film-coated ITO as the catalytic electrode to image the H_2_ and O_2_ nanobubbles during overall water splitting [57]. They demonstrated that on the Au/Pt film surface, O_2_ nanobubbles are detectable at the early stages of the OER, whereas the formation of H_2_ nanobubbles occurs only under a substantial overpotential.

In light of previous studies and leveraging an interdisciplinary approach, our group has recently integrated computer science, statistics, and nanotechnology expertise to address a critical problem in transition metal disulfide (TMD) catalysis [58]. Specifically, we employed a correlative integration of multi-techniques, including on-chip microcell devices, total internal reflection fluorescence microscopy platform (TIRF), and atomic force microscopy (AFM), as shown in Fig. 2D. This unique approach allows us to visualize the heterogeneity of local activity on MoS_2_ basal plane with a sulfur vacancy concentration of 6.6%, and generate a corresponding 3D strain map. By systematic image correlation and complex statistical analyses, we demonstrated strain effectively activates sulfur vacancies on the MoS_2_ basal plane, with higher strain correlating with increased local activity (Fig. 2E-F). Furthermore, we isolated the distinct effects of compressive and tensile strain on HER activity, revealing that tensile strain has a remarkably greater enhancing effect on activity than the compressive part (Fig. 2F-I).

**Fig. 3:**
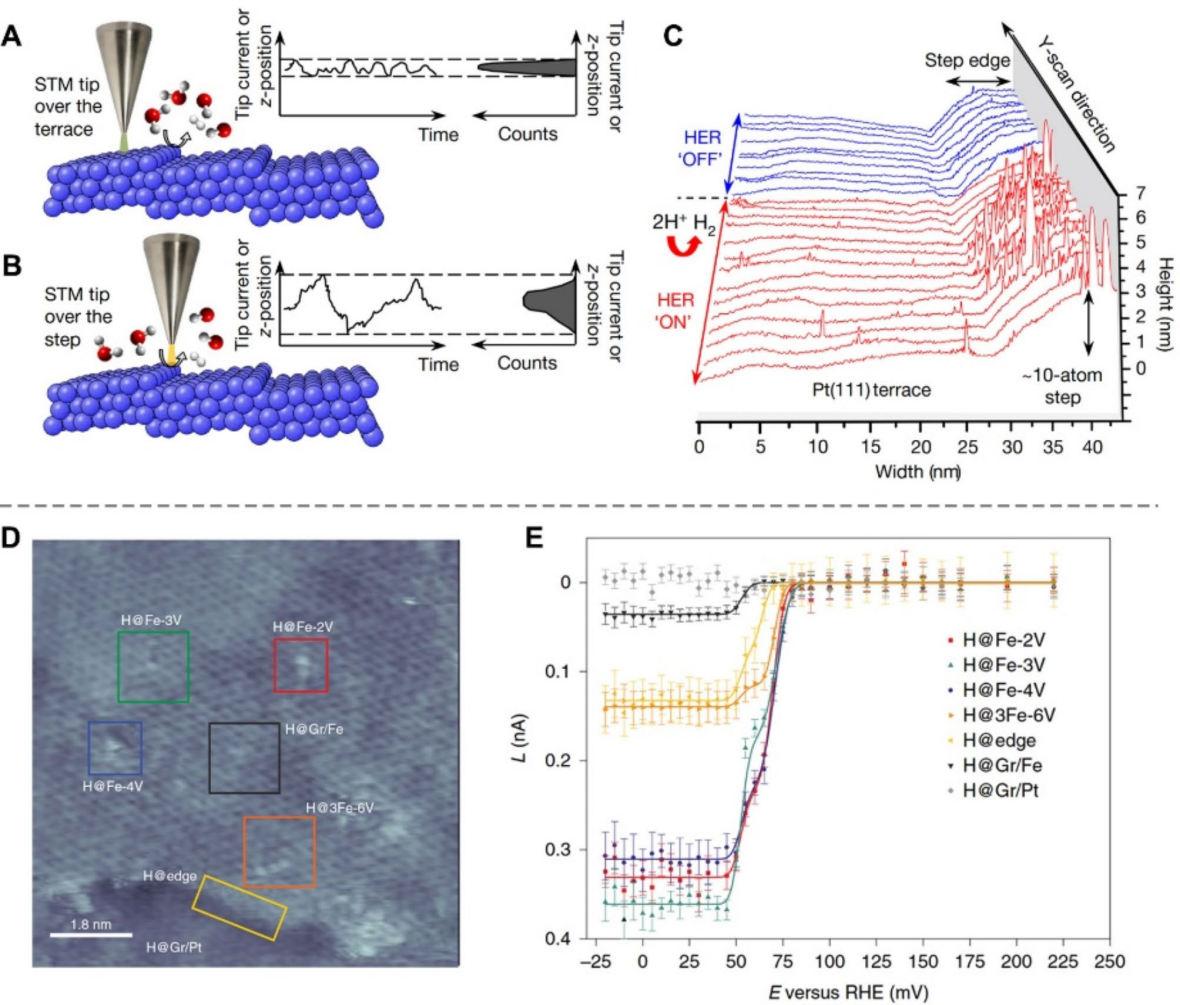
Atomic-scale visualization of active sites using EC-STM technique. (**A**-**B**) Schematic diagram of EC-STM principles, showing that the amplitude of the tunneling-current noise is proportional to the reactivity of surface sites. (**C**) EC-STM line scans (constant-current mode) obtained over a Pt(111) surface in 0.1 M HClO_4_. Reproduced with permission from [26]. Copyright 2017, Springer Nature. (**D**) EC-STM topographic images of the Gr/Fe (1.8monolayer)/Pt(111) surface recorded at E = 195 mV. STM image showing the different structural units investigated, Fe-2 V, Fe-3 V, Fe-4 V, 3Fe-6 V. (**E**) Normalized current roughness, L, as a function of E extracted from the areas outlined by the rectangular boxes in D. Reproduced with permission from [10]. Copyright 2021, Springer Nature

To further differentiate active sites at the atomic level, EC-STM has been developed to leverage the high spatial resolution capabilities of traditional STM while simultaneously obtaining electronic signals that reflect catalytic reactivity. Specifically, the STM tip collects both the height and tunneling current between the STM tip and the sample surface (Fig. 3A). Importantly, the noise from the tunneling current reflects site-specific electrocatalytic activity (Fig. 3A-B), enabling precise active site identification (Fig. 3C) [26]. For instance, the Granozzi group utilized operando EC-STM to investigate the electrochemical activity of zero-dimensional (0D) Fe single atoms at the graphene interface during HER [10]. The *L*(*E*) curves for different sites (Fig. 3D) reveal that the most active sites are iron atoms trapped in vacancy clusters (H@Fe-2 V, H@Fe-3 V, and H@Fe-4 V, where H@Fe-2 V, H@Fe-3 V, and H@Fe-4 V represent hydrogen adsorption at an iron atom trapped by 2, 3, and 4 carbon vacancies, respectively). These are followed by the iron step edge (H@edge) and the iron basal plane covered by graphene (H@Gr/Fe), as shown in Fig. 3E.

Similarly, J. Rost and colleagues used operando EC-STM to study the relationship between the evolution of electrochemical signals and surface roughening of Pt(111) [59]. They proposed that the surface of Pt(111) exhibited a “nucleation and early growth—late growth” regime, where the early stages contributed to both electrochemical activity and roughness evolution, while the latter stages primarily influenced roughness alone.

Although operando EC-STM offers high spatial resolution, it has intrinsic limitations: (i) The catalysts must exhibit good electrical conductivity, which limits the technique’s applicability to semiconducting electrocatalysts [60]. (ii) The catalysts should have a relatively flat surface and a well-defined structure. (iii) Limited temporal resolution, ranging from milliseconds to seconds, is insufficient to capture the electrocatalytic dynamics, which occur on the order of picoseconds to femtosecond (∼ ps to fs) [61].

### Reaction mechanisms and pathways

Heterogenous catalytic reactions often involve multiple steps, with a rate-determining step that significantly influences overall reaction efficiency—akin to the “buckets effect”. In other words, the efficiency of catalytic reactions is largely determined by this rate-determining step. To this end, researchers have focused on “tracking” the reaction by probing the electronic configuration and local chemical environment of active sites using operando techniques [62, 63]. These approaches provide valuable information about the formation of reactant-catalysts bonds, the generation of intermediates, the evolution of the chemical states of the catalyst throughout the reaction process, and so on.

**Fig. 4:**
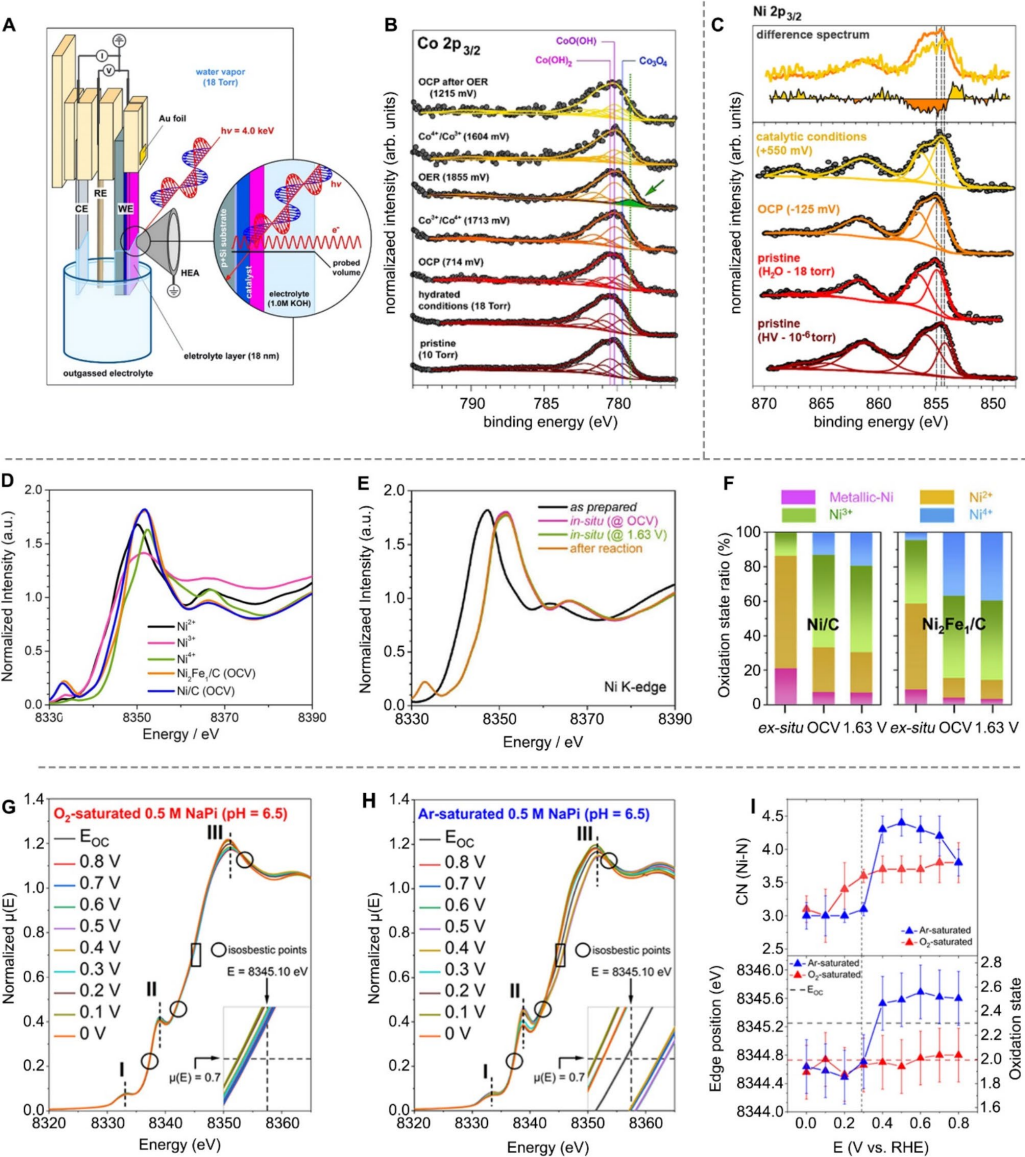
Investigation of reaction mechanism using the operando XAS and XPS. (**A**) Schematic illumination of the operando electrochemical AP-XPS experimental configuration. (**B**) Co 2*p*_3/2_ XPS spectra recorded under hydrated conditions (18 Torr water vapor) as a function of applied electrochemical potential, showing dynamic changes in oxidation state. Reproduced with permission from ref [66]. Copyright 2017, American Chemical Society. (**C**) Operando AP-XPS spectra of the Ni 2*p*_3/2_ photoelectron peaks under varied conditions, illustrating the effect of the environment on Ni oxidation states. Reproduced with permission from ref [67]. Copyright 2017, American Chemical Society. (**D**) Ni K-edge XANES spectra of the Ni/C and Ni_2_Fe/C samples at the open-circuit voltage (OCV) state, with the reference spectra of Ni^2+^, Ni^3+^, and Ni^4+^ samples. (**E**) Operando XANES spectra collected at the Ni K-edge for Ni_2_Fe_1_/C. (**F**) Oxidation-state distribution for Ni/C and Ni_2_Fe_1_/C, deconvoluted using a linear combination method. Reproduced with permission from ref [69]. Copyright 2022, Cell Press. (**G-H**) Operando XANES spectra of Ni − HAB/carbon fiber paper (CFP) under O_2_-saturated (**G**) and Ar-saturated (**H**) electrolytes during the ORR process, highlighting differences in electronic structure. (**I**) Coordination numbers extracted from Extended X-ray Absorption Fine Structure (EXAFS) (upper panel) and XANES Ni K-edge positions (bottom panel) for Ni − HAB/CFP under O_2_- and Ar-saturated conditions. Reproduced with permission from ref [14]. Copyright 2022, American Chemical Society

For instance, operando ambient-pressure XPS (AP-XPS) can probe the valence information of catalytic sites with a penetrated depth of 10–30 nm in the liquid phase and 2–10 nm in the solid phase [64]. It enables the real-time investigation of the interplay between the chemical/electronic structure of catalysts, the electrochemical environment, and reaction intermediates or products [15, 65]. A notable instance of this application is the work by Sharp and co-workers, who used operando AP-XPS to explore the OER mechanism on CoO_x_ surfaces (Fig. 4A) [66]. Their study revealed that dynamic changes in oxidation states and surface hydroxylation under reaction conditions enhanced the OER activity of CoO_x_ catalysts, providing critical insights into the roles of active species and intermediates in catalytic efficiency (Fig. 4B). In another application, Yano and colleagues employed operando AP-XPS to study the surface chemistry of a multicomponent Ni_0.3_Fe_0.07_Co_0.2_Ce_0.43_O_x_ catalyst under OER conditions (Fig. 4C) [67]. Their findings highlighted the oxidation of Ni from Ni(II) to Ni(III), leading to the formation of the active Ni(III)O(OH) phase during catalysis, which is crucial for enhancing catalytic efficiency.

Compared to XPS, XAS is widely employed to understand the coordination environments and oxidation states of elements in catalysts with much deeper penetration depth, which ranges from hundreds of nanometers to several microns, depending on the X-ray energy and the angle of incidence relative to the sample [68]. For example, Oh and colleagues employed the synchrotron-based operando X-ray absorption near edge structure (XANES) analysis to monitor the oxidation states of Ni species in Ni_2_Fe_1_ LDH (layered double hydroxide) [69]. During OER, Ni (pristine Ni^2+^/^3+^) species tend to transition to higher oxidation states (Ni^3+^/^4+^) in both Ni/C and Ni_2_Fe_1_/C samples, as shown in Fig. 4D-E, which can be attributed to the formation of active β- and γ-NiOOH [70–72], as evidence by ex-situ XRD analysis. Notably, XANES spectral comparisons indicate that this transformation is chemically induced before electrochemical reactions occur (Fig. 4E). As shown in Fig. 4F, the Ni^4+^ ratio in the Ni_2_Fe_1_/C sample is higher than that in the Ni/C sample, indicating that the presence of Fe species promotes the oxidation of low-valence Ni species in alkaline media. More importantly, these oxidation state modifications are imperative to the Mars-Van Krevelen mechanism [73], whereby Ni^3+^/^4+^ redox reaction facilitates OER through deprotonation and formation of stabilized intermediates with low free-energy barriers for OH* to O* oxidation, significantly enhancing the reaction rate.

In another case study, Jin’s group utilized operando XAS to examine the oxidation state dynamics of Ni in 2D Ni_3_HAB_2_ (HAB = hexaaminobenzene) metal-organic frameworks during the ORR (Fig. 4G-H) [14]. They found that in Ar-saturated electrolytes, cathodic potentials triggered dynamic changes in the Ni oxidation state, while O_2_-saturated conditions caused negligible variation. Their analysis suggested that the two-electron ORR mechanism predominates when E > E_redox_ (∼ 0.3 V_RHE_), while Ni-mediated linker discharge and electrocatalytic interactions dominate at E < E_redox_ (Fig. 4I).

**Fig. 5:**
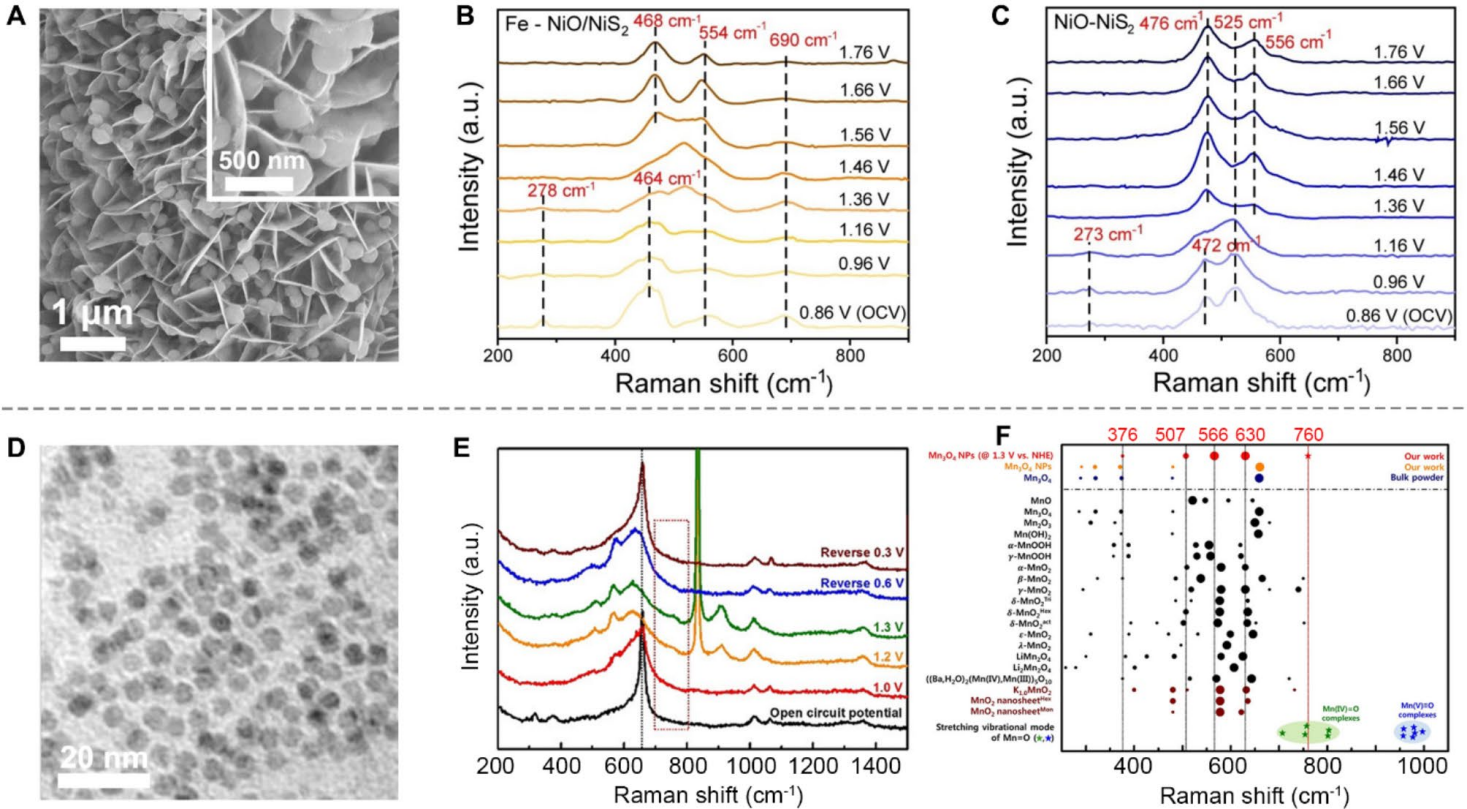
Monitoring the reaction intermediates using operando Raman spectroscopy. (**A**) SEM images of Fe NiO/NiS_2_. (**B**-**C**) Potential-dependent operando Raman spectra of Fe NiO/NiS_2_ (**B**) and NiO/NiS (**C**). Reproduced with permission from ref [75]. Copyright 2022, Wiley. (**D**) TEM image of Mn_3_O_4_ NPs with the size of 4 nm. (**E**) Potential-dependent operando Raman spectra with increasing applied potential in 1 M KHCO_3_ electrolyte. (**F**) Analysis of Raman peak positions. Reproduced with permission from ref [16]. Copyright 2021, Wiley

Identifying catalytic sites provides valuable insights into which types of sites exhibit the highest intrinsic activity. Yet, to fully comprehend critical catalytic processes, it is essential to monitor reaction intermediates involved in multi-step, complex reactions (e.g., CO_2_ RR, ORR, and OER). Examining these intermediates reveals the stepwise mechanisms that link the electronic properties of catalysts with their performance, delivering profound insights into the dynamics of catalytic processes [74]. For example, Yan and co-workers employed operando Raman spectroscopy to investigate dynamic surface reconstruction and sulfur oxidation corrosion behavior in 2D Fe-doped NiO/NiS_2_ catalysts during the OER (Fig. 5A) [75]. They reported that Fe doping significantly influenced the electrochemical behavior of the catalysts. Specifically, Ni metal sites in Fe-NiO/NiS_2_ were more readily oxidized to NiOOH at OCV compared to the pristine Ni/NiS_2_ counterpart (Fig. 5B). Additionally, the Ni-S characteristic peak in Ni/NiS_2_ disappeared at 1.16 V. In contrast, in Fe-Ni/NiS_2_, this peak persisted until 1.36 V (Fig. 5C), indicating that Fe doping effectively mitigated sulfur corrosion under OER conditions.

In another study, Nam et al. utilized operando Raman spectroscopy to identify active intermediates, specifically Mn-oxo moieties, during OER in 0D Mn_3_O_4_ nanocatalysts (Fig. 5D) [16]. Their analysis pinpointed a vibration peak at 760 cm⁻¹, attributed to high-valent Mn^4+^=O species. This assignment was confirmed by comparing the Raman band position and isotopic shifts with previously reported data (Fig. 5E–F).

While operando Raman spectroscopy provides valuable insights into the structural and chemical evolution of catalysts, its application faces limitations due to the inherently weak Raman signals in electrolytes, where scattering by solvent molecules reduces signal intensity [17]. Surface-enhanced Raman spectroscopy (SERS), driven by surface plasmon resonances, is a powerful method for enhancing Raman signals and achieving high signal-to-noise ratios [76]. The substantial SERS effect requires specified metal substrates with roughened surfaces or in the form of nanoparticles [77]. However, its applicability is limited by several inherent drawbacks: (i) Bare nanoparticles are directly exposed to the chemical environment (i.e., liquid or gaseous), leading to the adsorption of matrix species such as solvent molecules (e.g., H_2_O, ethanol), reaction by-products (e.g., carbonates), or electrolyte ions (e.g., Cl^−^, SO_4_^2−^), which can contribute to background Raman signals and introduce inaccuracies. (ii) Charge transfer may occur between metal nanoparticles and other metallic substrates due to differences in their Fermi levels. This interaction can interfere with the intrinsic catalytic properties of the material under study [78]. (iii) Target molecules, such as OH^−^ and H_2_O, potentially interact with bare nanoparticles, for example, altering their electron density and adsorption configurations. These factors can cause significant changes in spectral features, leading to misinterpretation of the spectra.

**Fig. 6:**
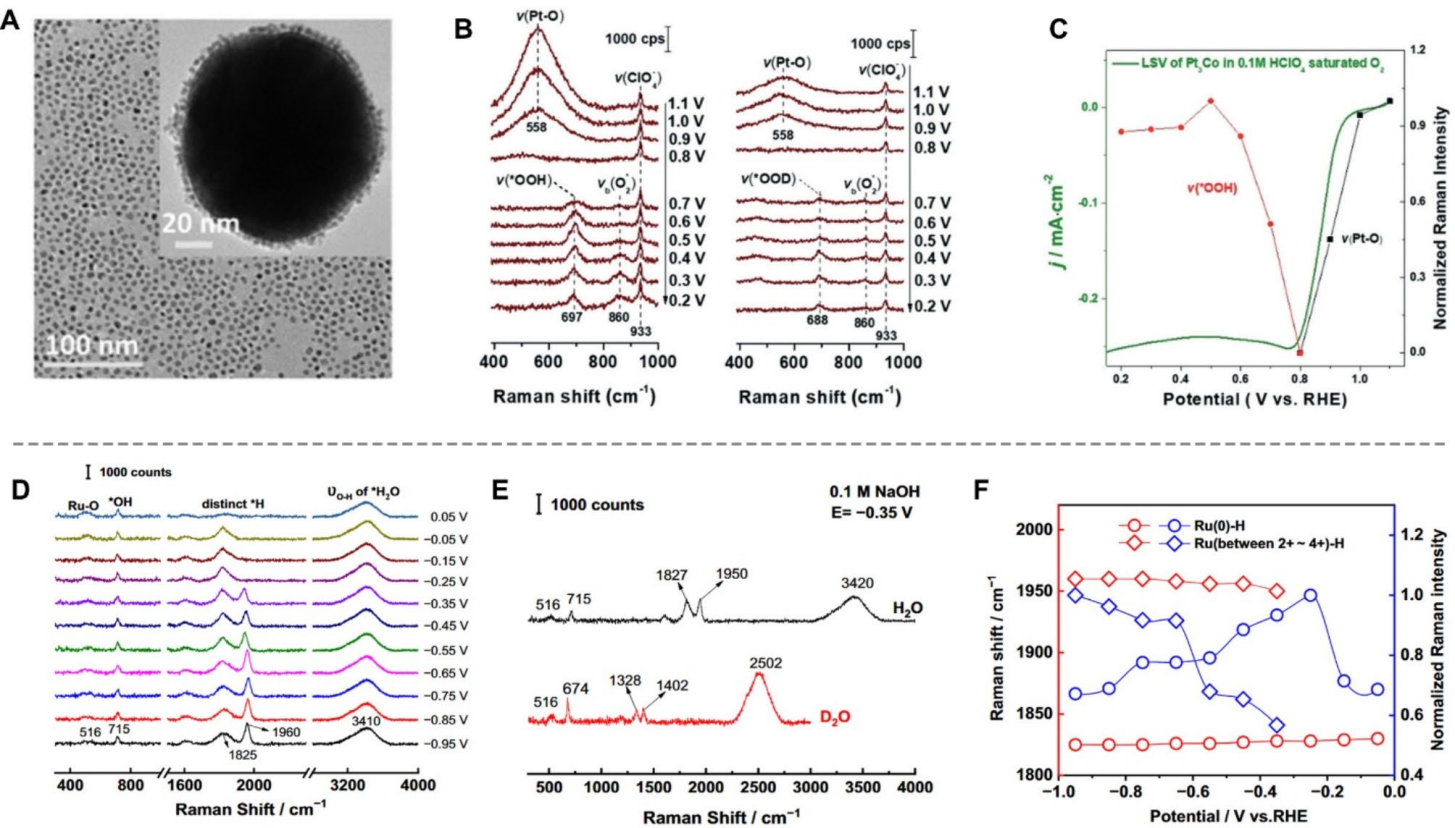
Monitoring the reaction intermediates using operando SERS spectroscopy. (**A**) TEM image of Pt_3_Co nanoparticles and Pt_3_Co-on-SHINs satellite nanocomposites. (**B**) Operando electrochemical-SHINERS (EC-SHINERS) spectra of dealloyed Pt_3_Co nanocatalysts in 0.1 M HClO_4_ with H_2_O and D_2_O solution saturated O_2_. (**C**) Normalized Raman intensities of Pt − O (black square) stretching mode, and *OOH (red sphere) at different potentials. Reproduced with permission from ref [81]. Copyright 2019, Wiley. (**D**) Raman spectra of 55 nm Au@2.5 nm Ru surface under various alkaline HER potentials. (**E**) Raman spectra of 55 nm Au@2.5 nm Ru surface at -0.35 V in different electrolytes. (**F**) Normalized Raman intensities (blue) and frequency shifts (red) of the Ru-H band at low-valence state Ru(0) (circle) and high-valence state Ru (between + 2 and + 4) (square) surfaces as a function of potential. Reproduced with permission from ref [82]. Copyright 2023, Springer Nature

In stark contrast, shell-isolated nanoparticle-enhanced Raman spectroscopy (SHINERS) overcomes these limitations by utilizing ultra-thin silica shells to isolate plasmon-active Au cores [77, 79, 80]. This approach has several advantages relative to SERS in studying electrocatalytic reactions under operating conditions. First, SHINERS can sustain harsh chemical environments owing to the chemical-inert silica protective shell. Second, the silica shell prevents direct interface contact between the Au core and catalyst, eliminating charge transfer. Third, Raman signals are generated purely from the target surface or molecules, without affection from external factors. Finally, the shell-isolated structure offers superior stability and durability compared to bare nanoparticles. For instance, Li and colleagues innovatively utilized the SHINES to study electrocatalytic ORR intermediates on Pt_3_Co nanocatalysts [81]. As shown in Fig. 6A, with this technique, they directly observed *OOH intermediates during ORR and verified their potential-dependent behavior (Fig. 6B and C). This demonstrated the utility of SHINERS in real-time tracking of reaction intermediates under electrocatalytic conditions.

Similarly, Li et al. employed core-shell nanoparticle-enhanced Raman spectroscopy with 55 nm Au@2.5 nm Ru core-shell nanoparticles to investigate interfacial water behavior and key intermediates on Ru surfaces during the HER process [82]. Their operando Raman analysis revealed a dual composition of the catalyst, comprising Ru(0) and a high-valent RuO_x_ shell (Fig. 6D). The high-valent Ru(n⁺) surfaces facilitated enhanced interactions, driven by the local cation tuning effect of Na·H_2_O and the elevated work function of Ru(n⁺) species. These factors promoted interfacial water dissociation and moderated adsorption energies for interfacial water, *H, and *OH, optimizing the HER process (Fig. 6E-F).

**Fig. 7:**
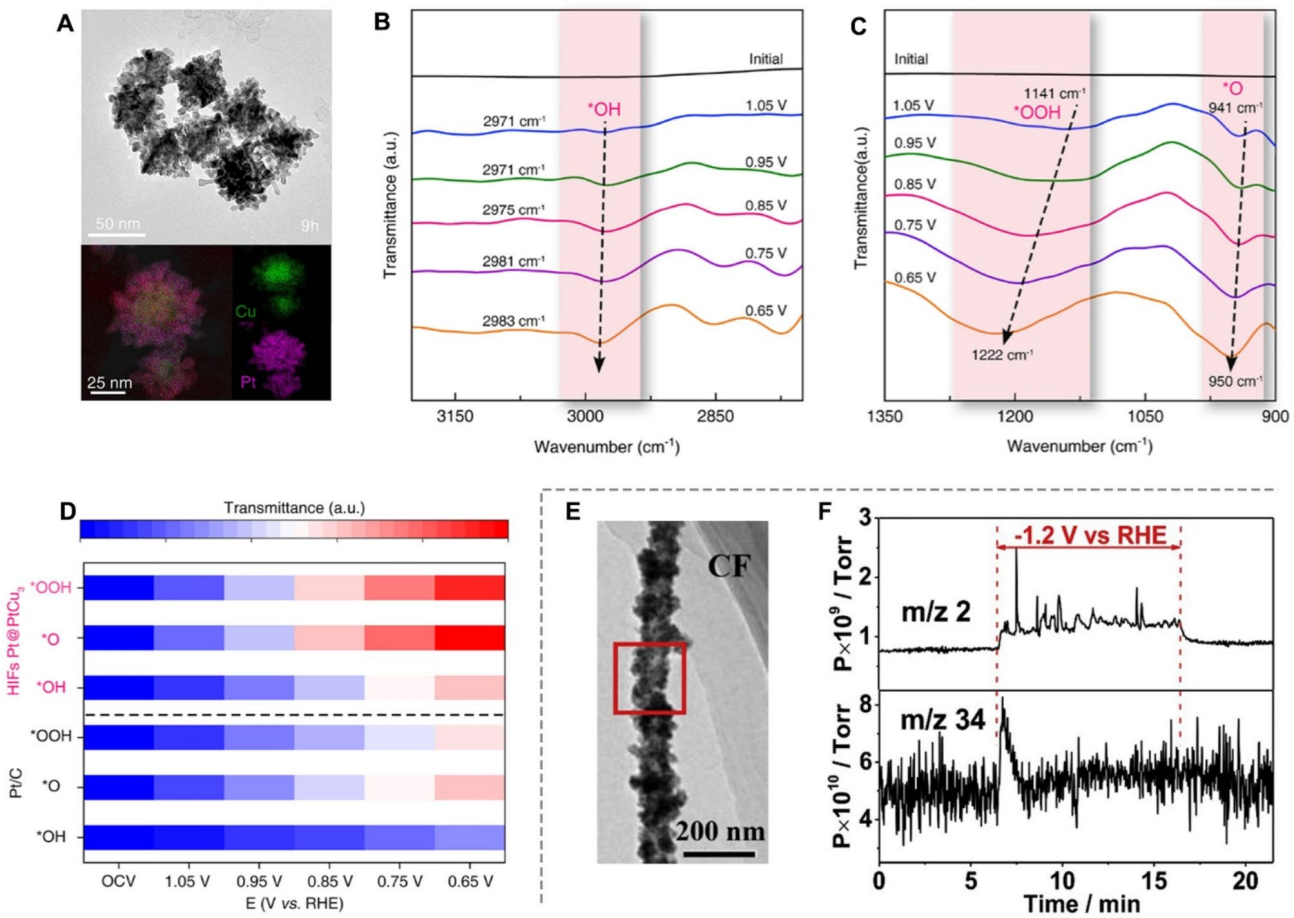
Investigation of intermediates through the IR spectroscopy and online electrochemical mass spectrometer. (**A**) Typical TEM image and corresponding energy-dispersive X-ray (EDX) mapping of HIFs Pt@PtCu_3_. (**B**-**C**) In-situ SRIR spectra for *OH (**B**), and *O as well as *OOH (**C**). (**D**) Intensity differences of ORR intermediates at HIFs Pt@PtCu_3_ and Pt/C. Reproduced with permission from ref [18]. Copyright 2024, American Chemical Society. (**E**) High-resolution TEM image of CdS-CNTs. (**F**) Operando DEMS of H_2_ and H_2_S during CO_2_RR. Reproduced with permission from ref [84]. Copyright 2019, Elsevier

IR spectroscopy utilizes infrared light to excite molecular vibrations, enabling the identification of compounds and reaction intermediates through infrared absorption spectra. Unlike Raman spectroscopy, IR spectroscopy excels in detecting intermediates with polar functional groups, such as hydroxyl (OH), carbonyl (C = O), and amino (NH) groups, due to its high sensitivity to molecular polarity. For instance, Yao et al. employed operando synchrotron radiation infrared (SRIR) spectroscopy to monitor reaction intermediates during the ORR on a 0D high-index Pt shell encapsulated PtCu_3_ core nanocatalyst (HIFs Pt@PtCu_3_) (Fig. 7A) [18]. Their analysis revealed three characteristic peaks corresponding to the stretching vibrations of *OOH, *O, and *OH intermediates (Fig. 7B-C). Importantly, these intermediates were more pronounced on the HIFs Pt@PtCu_3_ catalyst than on a Pt/C benchmark, indicating enhanced ORR kinetics due to the high-index Pt structure (Fig. 7D).

Online DEMS complements IR spectroscopy by detecting volatile intermediates and validating reaction mechanisms [83, 84]. Peng and colleagues used operando DEMS to study the formation of sulfur (S) vacancies in a 1D CdS-CNTs catalyst during the electrocatalytic CO_2_RR (Fig. 7E) [84]. Their findings showcased that at an applied potential of -1.2 V_RHE_, hydrogen sulfide (H_2_S) gas intermediates were detected (Fig. 7F). This result indicates that adsorbed hydrogen reacts with S^2^⁻, creating sulfur vacancies on the catalyst surface, which in turn facilitates the generation of CO products.

### Product formation

The determination of reaction products allows one to specify the reaction pathway and further shed light on the critical catalytic process. Online DEMS is a powerful operando tool for analyzing gaseous products in real-time during electrochemical reactions. The technique couples an electrochemical cell with a mass spectrometer through a gas-permeable membrane, which selectively allows volatile species to diffuse into the mass spectrometer. Inside the mass spectrometer, these species are ionized, and their mass-to-charge ratios are analyzed to identify and quantify them [85]. The resulting data can be synchronized with electrochemical parameters such as applied potential or current, enabling the direct correlation of product formation with reaction kinetics and mechanisms.

**Fig. 8:**
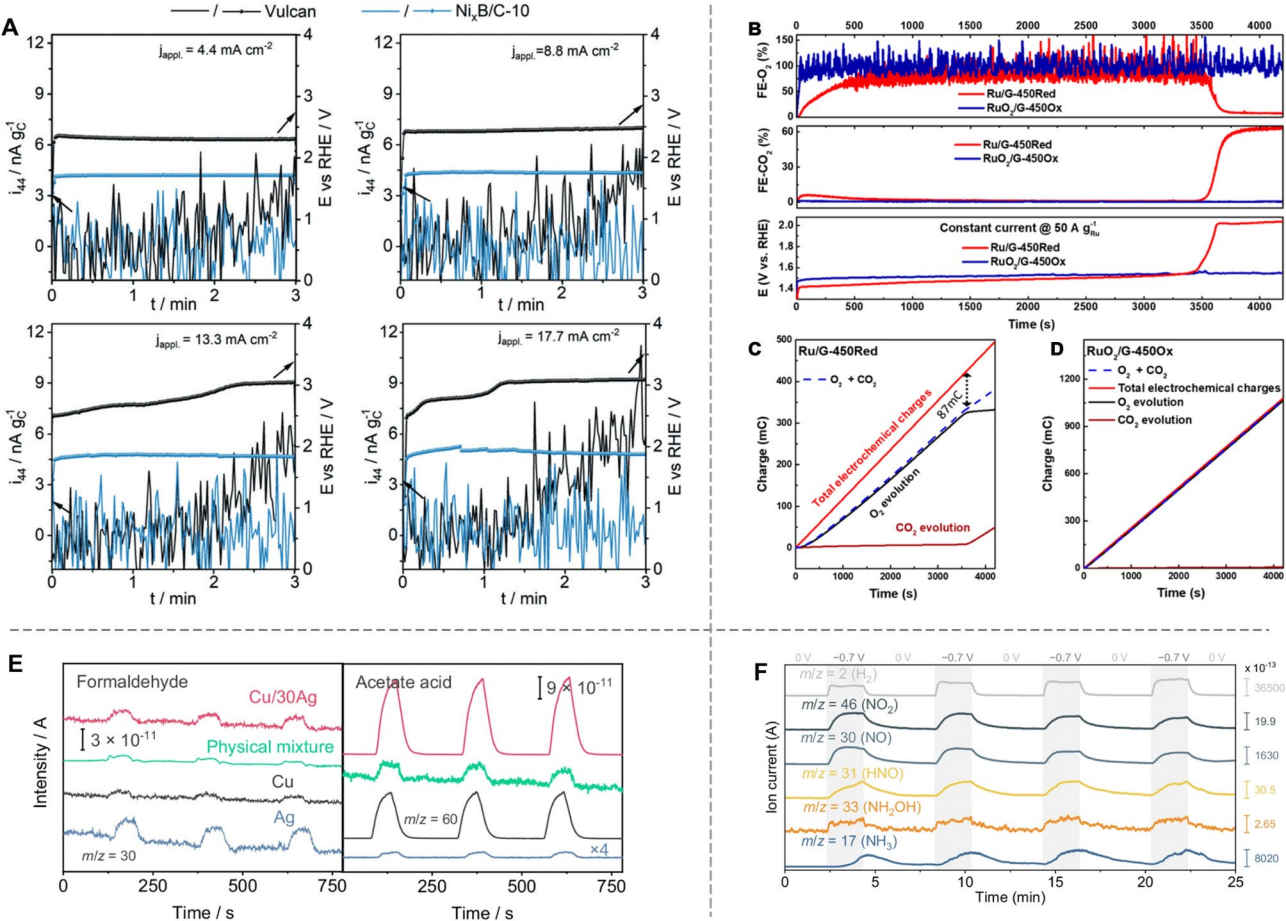
Investigation of product formation by online EC mass spectroscopy. (**A**) Chronopotentiometric measurements and ion currents for CO_2_ in Vulcan and Ni_x_B/C-10 catalysts at various current densities. Reproduced with permission from ref [86]. Copyright 2020, Wiley. (**B**) Faraday efficiency for CO_2_ evolution (FE-CO_2_), O_2_ evolution (FE-O_2_), and electrode potential as a function of time at a constant current. (**C**-**D**) Total electrochemical and transferred charges for O_2_ and CO_2_ evolution of Ru/G-450Red (**C**) and RuO_2_/G-450O_x_ (**D**), respectively. Reproduced with permission from ref [87]. Copyright 2021, American Chemical Society. (**E**) Online DEMS measurements of Cu, Ag, Cu/30Ag, and the physical mixture during the CORR. Reproduced with permission from ref [88]. Copyright 2024, Springer Nature. (**F**) In-situ DEMS measurements for electrocatalytic NO_3_RR at − 0.7 V over four continuous cycles on ZnPc MDE. Reproduced with permission from ref [89]. Copyright 2024, Springer Nature

For instance, Schuhmann and colleagues introduced a novel DEMS approach to investigate CO_2_ production and deduce carbon corrosion kinetics [86]. Their study revealed that the OER-inert carbon black (Vulcan XC 72) exhibited significant CO_2_ generation, achieving a faradaic efficiency of 75–80% during chronopotentiometric testing. In contrast, no CO_2_ was detected with OER-active carbon-supported nickel boride catalysts (Fig. 8A), suggesting that the OER catalyst effectively protects carbon from oxidation in alkaline conditions.

Wen’s group further explored the electrocatalytic OER mechanism using graphene-supported Ru nanocatalysts. Employing real-time monitoring of catalytic products (O_2_ and CO_2_) via an online chip-based electrochemistry–mass spectrometry (chip EC-MS) system [87], they highlighted the critical role of pre-oxidation for Ru-based catalysts. This preparation was found to: (i) facilitate rapid O_2_ production (∼ 10 s) with 100% faradaic efficiency in the pre-oxidized RuO_2_/G-450O_x_ (graphene-supported RuO_2_ nanoparticles prepared by annealing reduction at 450 °C) catalyst, unlike the pristine Ru/G-450Red (graphene-supported Ru nanoparticles prepared by annealing reduction at 450 °C) counterpart (Fig. 8B and D); (ii) require ∼ 700 s for the Ru/G-450Red catalyst to achieve steady-state faradaic efficiency (∼ 79%), necessitating ∼ 2% (∼ 8.8 mC) of the total charge to oxidize metallic Ru to RuO_2_ and an additional 19% for partial oxidation of the graphene support (Fig. 8C); and (iii) exhibit greater OER deactivation in Ru/G-450Red due to persistent corrosion of the graphene support, evidenced by a marked increase in CO_2_ faradaic efficiency (Fig. 8B).

Selectivity is a critical factor in electrocatalysis, as it determines the efficiency and economic viability of desired product formation. In-situ DEMS has been widely utilized to detect and quantify reaction products, providing valuable insights into catalytic selectivity.

For example, Loh et al. employed DEMS to investigate the selectivity of a Cu-Ag co-catalyst system for the electrochemical conversion of CO to oxygenates [88]. By detecting key intermediates such as COH, formaldehyde (HCHO), and acetic acid, they demonstrated that the Cu/30Ag (mole ratio of Cu to Ag is 7:3) catalyst exhibited the strongest DEMS signal for acetic acid, indicating superior selectivity toward oxygenates compared to pure Cu, pure Ag, or physically mixed Cu and Ag systems (Fig. 8E). These results further revealed that pure Ag primarily produced formaldehyde. In contrast, the Cu/30Ag system facilitated the rapid transfer and conversion of COH intermediates at the Ag/Cu interface, enhancing oxygenate formation while suppressing ethylene production and the hydrogen evolution reaction.

Similarly, Tang et al. utilized DEMS to analyze intermediates in nitrate reduction (NO_3_RR) on a zinc phthalocyanines (ZnPc) molecularly dispersed electrocatalysts (MDE) catalyst [89]. By tracking mass-to-charge ratios (m/z) in a 1.0 M KOH + 1.0 M KNO_3_ solution, they identified hydroxylamine (NH_2_OH) as a key intermediate in the reaction pathway (Fig. 8F). Multi-potential step tests revealed that mass signals for NH_2_OH and other species (e.g., NO_2_, NO, NH_3_) fluctuated dynamically with applied potential, providing direct evidence of selective catalytic pathways. These findings confirmed that under specific conditions, the ZnPc MDE catalyst favors NH_2_OH formation over NH_3_, shedding light on reaction mechanisms that regulate product selectivity.

In summary, we have reviewed and discussed recent advancements in in-situ experimental techniques for investigating key electrocatalytic processes, such as identifying active sites, elucidating reaction mechanisms and pathways, and monitoring product formation. Techniques like SECM, SECCM, single-molecule fluorescence microscopy (SMFM), and EC-STM excel in pinpointing active sites with nanometric to atomic-scale precision. Meanwhile, in-situ methods like AP-XPS, XAS, Raman, SERS, IR, and online DEMS can determine reaction mechanisms and pathways, with online DEMS specifically adept at monitoring product formation during electrocatalytic reactions. Table 1 provides a comprehensive overview of the advantages, disadvantages, spatial resolutions, and temporal resolutions of the above in-situ experimental techniques, aiding researchers in assessing their suitability for various applications.

**Table 1:** Summary of Operando techniques introduced in this work

Technique	Advantages	Disadvantages	Spatial Resolution	Temporal Resolution
Operando Raman	Real-time analysis of dynamic surface reconstructions	Weak signal in aqueous environments	∼ 1 μm	~ms to s
	Molecular-level insights	Limited sensitivity to low concentrations		
	Non-destructive			
SERS/SHINERS	Enhanced signal by ∼ 8 orders	Complex nanoparticle fabrication	~nm level	~ms to s
	Operando detection of intermediates	Surface-dependent enhancement		
	Broad substrate compatibility			
IR Spectroscopy	Sensitive to polar intermediates	Weak signals from non-polar groups	∼ 10 μm	~ms to s
	Identifies functional groups (e.g., OH, C = O)	Overlapping spectral features		
	Broad applicability			
DEMS	Real-time detection of volatile intermediates	Limited to gaseous/volatile products	N.A.	~ms
	Mechanistic validation	Membrane durability issues		
	Broad reaction applicability			
Operando XAS	Probes electronic structure and coordination environment	Primarily bulk-sensitive	~nm	~ms to s
	Captures bulk and active site dynamics	Requires synchrotron access		
	High versatility			
AP-XPS	1. Surface-sensitive (2–10 nm solid phase)	Limited depth probing	~nm	~s
	Monitors surface chemistry in real-time	Complex setup and calibration		
	Operando capabilities			
EC-STM	Atomic-scale visualization	Requires flat, conductive surfaces	~Å	~ms to s
	Identifies active sites precisely	Limited temporal resolution		
	High spatial resolution			
SECM	Maps surface activity with high-resolution	Limited to electrochemical reactions	∼ 10–20 nm	~ms to s
	Pinpoint active sites	Slower scanning speed affects the resolution		
	Operando monitoring			

## Data mining-driven catalyst design

Data mining refers to the systematic sampling and analysis of large-scale datasets to discover rules, trends, and hidden knowledge for specific research objectives, utilizing methods from machine learning (ML), statistics, and database systems [90]. Recently, this methodology has emerged as a transformative approach in electrocatalysis, facilitating the extraction of statistical insights and patterns relating to material optimization, synthesis, and catalytic properties [91–93]. Specifically, targeting a particular type of reaction or catalyst, data mining approaches can first quantitively “learn” relationships between factors in the database and desired properties, and then leverage the learned knowledge to screen the optimized materials, or predict the outcome of unseen materials, or even generate new and valid catalyst structures.

This section will begin by introducing the systematic protocols and providing an overview of the data mining process. It will then present examples of how data mining methods facilitate informed decision-making in catalyst design, emphasizing the integration of domain knowledge throughout the process.

### Typical workflow

**Fig. 9:**
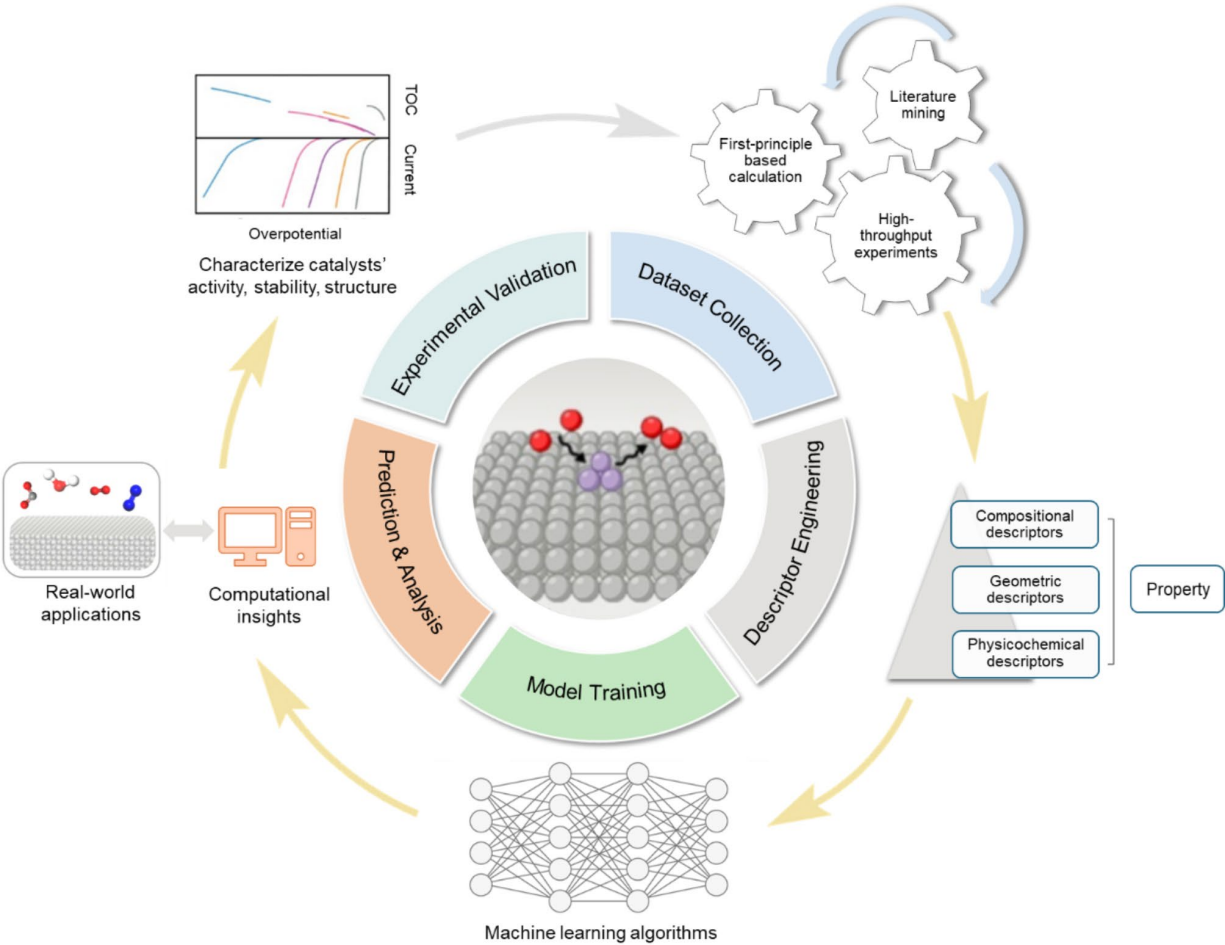
Typical workflow of data mining-driven catalyst design

A typical data-mining-driven catalyst design process primarily involves five basic steps: dataset collection, descriptor engineering, model training and validation, prediction and analysis, and experimental validation (Fig. 9). While extensive reviews are available on comprehensive data mining or ML concepts [94–96], this section will provide a concise overview of the five fundamental steps, focusing on their specific applications in catalysis research.

First, dataset collection can be achieved through high-throughput experiments (HTE) [97, 98] or first-principles-based calculations [99–101], which rapidly screen multiple catalyst formulations to generate extensive datasets, or through literature mining [90, 102], which involves extracting relevant information from existing scientific publications. It is worth mentioning that HTE platforms can integrate synthesis, characterization, and performance evaluation, even using advanced robotic equipment. Notable examples include Autonomous Laboratory for Inorganic Powder Synthesis (A-Lab) [103], Robotic Assistant for Automated Chemistry Experimentation and Characterization (ORGANA) [104], mobile robotic chemist [97, 105] and so on. For instance, A-Lab autonomously synthesizes novel inorganic compounds by integrating computational methods, ML, and robotics [103]. Such a system employs graph neural networks trained on computational materials databases and natural language models to propose initial synthesis recipes, which are then optimized through active learning based on thermodynamic data. Over 17 days, it successfully synthesized 41 out of 58 targeted novel compounds, primarily oxides and phosphates.

Second, carefully selecting and engineering appropriate descriptors are critical for developing accurate predictive models. Descriptors are chosen based on domain knowledge; specifically, their potential correlation with key performance indicators, such as turnover frequency (TOF), exchange current density, selectivity, and overpotentials. These features can be roughly categorized into three groups: compositional (for example, atomic number, atomic radius, electronegativity, valence orbitals, ionization energy), geometric (such as morphology, nanoparticle size, surface area, pore size), and physicochemical descriptors (such as binding energy, free adsorption energies [106], band gaps, and d-electron center position), which can be obtained from experiments, first-principles based calculations, and crystallography. Readers may refer to other comprehensive reviews for a detailed overview of typical descriptors [91, 107–109].

Third, appropriate ML algorithms—supervised and unsupervised learning—are employed to identify patterns between descriptors and target properties [96]. Compared to expert approaches, ML can reduce human bias that may arise from limited databases or subjective interpretations. By mining extensive datasets, these algorithms uncover quantitative patterns rather than personal judgment, leading to more objective and consistent decision-making and predictions in catalyst design. Common supervised ML models include Decision Trees (DT), Neural Networks (NN), Support Vector Algorithms, and Gaussian Process Regression (GPR) [96]. These models use a loss function to quantify the difference between predicted outputs and actual target values from the training dataset, optimizing model parameters by minimizing this loss function to achieve high predictive accuracy. In parallel, unsupervised learning—which seeks to extract patterns in unlabeled datasets—is valuable for uncovering hidden insights that may be useful for establishing predictive correlations. Frequently used unsupervised learning algorithms include clustering and dimensionality reduction methods [96].

Fourth, prediction and analysis serve as the bridge between computational insights and real-world applications. Once ML models are trained and validated, they are applied to predict the targeted properties—such as catalytic activity, stability, and selectivity—of unexplored catalysts. In addition, further statistical analysis can provide valuable mechanistic insights. For example, sensitivity analysis and feature importance metrics can assist in identifying the most influential descriptors affecting catalytic properties [110–112]. When coupled with first-principles calculations, this approach can reveal the underlying factors that govern catalyst performance.

In the last step, the predicted catalysts are synthesized and tested, where HTE setups are often employed to expedite the evaluation of multiple candidates. For example, robotic systems and automated testing platforms can characterize catalysts’ activity, stability, and structural properties, providing a direct comparison with computational predictions. Furthermore, discrepancies between experimental results and model predictions are used to refine the dataset and model. This iterative process—often referred to as “active learning”—enables continuous improvement in prediction accuracy. Experimental feedback may also uncover unforeseen phenomena, leading to new hypotheses and further advancements in understanding catalyst behaviors. Ultimately, the synergy between prediction and experimental validation ensures a robust, efficient pathway for the discovery and optimization of next-generation catalysts. As a typical example, Zhou et al. applied data-driven and HTE strategies to accelerate the discovery of high-entropy alloy (HEA) nanoparticle electrocatalysts for HER. With the assistance of THE and ML algorithms, two compositions of HEA are recommended, i.e., Fe_0.15_Co_0.4_0Ni_0.05_Pt_0.32_Pd_0.08_ and Fe_0.15_Co_0.40_Ni_0.05_Pt_0.28_Pd_0.12_., which are later validated by macro-electrochemical measurements [113].

### The incorporation of domain knowledge in data-mining catalyst design

Black-box data-mining algorithms often struggle with results interpretability and generality when applied to complex data sets beyond the given data. This limitation can be significantly alleviated by incorporating established scientific principles into data-mining algorithms. As mentioned earlier, some theoretical foundations have been established over the years, including Sabatier’s principle-based volcano plot [114, 115], electronic structure theories [116, 117], linear scaling relationships [118, 119] and Brønsted − Evans − Polanyi relation [120]. By applying these domain knowledge, data-mining techniques accelerate the identification of promising materials, reduce experimental costs, and foster innovation in catalyst design. This section will present implementation examples, ranging from introductory applications of physical meaning features to more sophisticated, in-depth analyses of design principles.

#### Domain knowledge integrated into feature selection

At the first level, domain knowledge is applied during the feature selection step. Since the goal is to map the correlation between input features and output targets, selecting features with relevant physical properties that potentially influence the targets can significantly enhance model accuracy. For instance, the smooth overlap of atomic positions (SOAP) and local density of states (LDOS) were selected to predict hydrogen adsorption strength on bimetallic nanoclusters [121]; Number of valence d electrons, electronegativity, metal-oxygen bond length, O 2*p* center, metal *d*-band center were used as descriptors to predict ORR onset potentials of non-precious transition metal-antimony oxides [122].

**Fig. 10:**
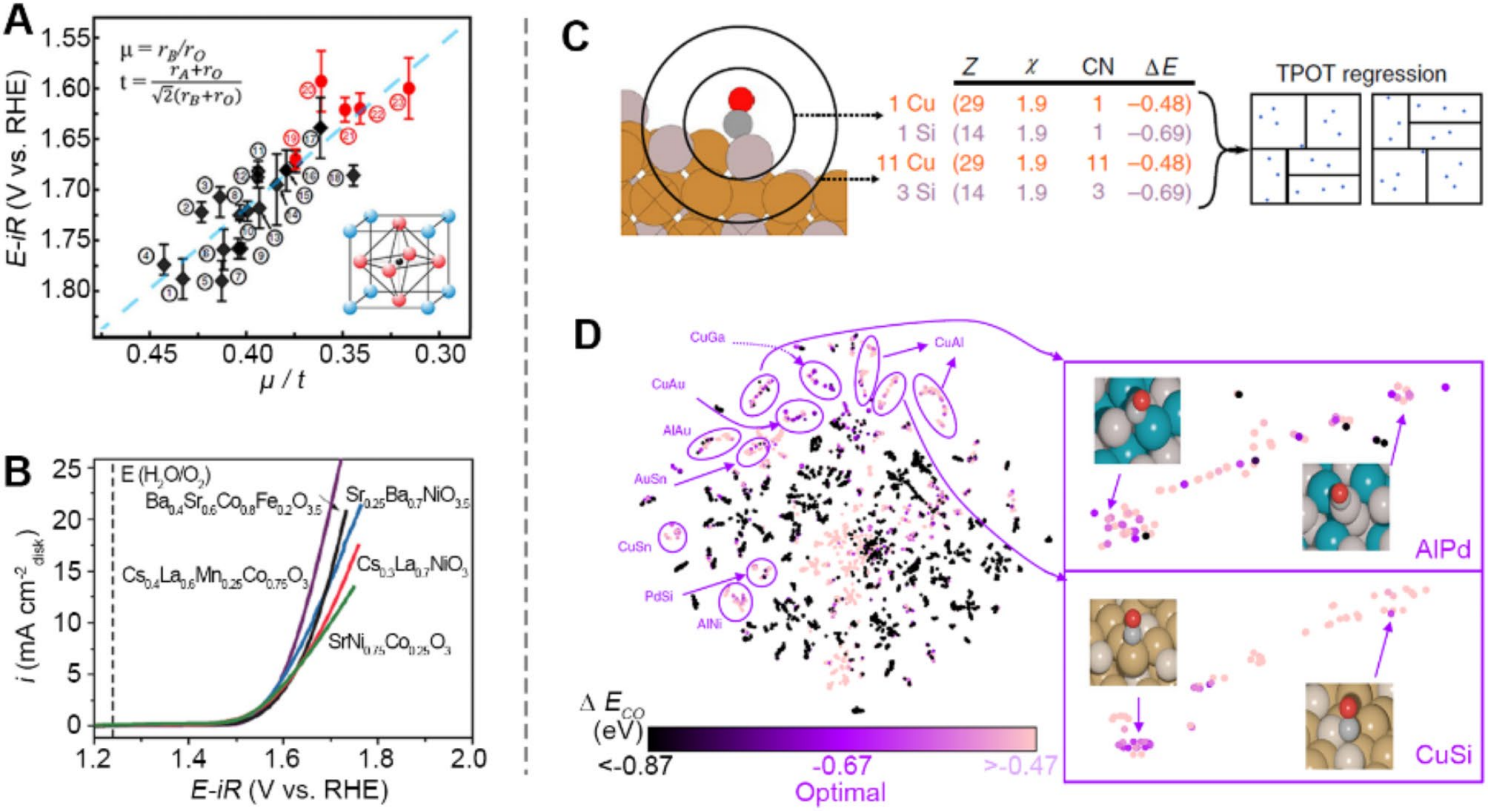
Examples of compositional and geometric descriptors being key features. (**A**) The linear relationship of overpotential and geometric descriptor *μ*/*t* for ABO_3_ oxide perovskites, where *μ* and *t* are the octahedral and tolerance factors, respectively. Inset is the schematic structure of the ABO_3_ perovskite, where the A, B, and O atoms are in black, red, and blue, respectively. (**B**) The linear sweep voltammetry (LSV) curves of four recommended oxide perovskites, including Cs_0.4_La_0.6_Mn_0.25_Co_0.75_O_3_, Cs_0.3_La_0.7_NiO_3_, SrNi_0.75_Co_0.25_O_3_, and Sr_0.25_Ba_0.75_NiO_3_. Reproduced with permission from ref [123]. Copyright 2020, Springer Nature. (**C**) The elemental descriptors for CO absorption sites on the surface of bimetallic electrocatalysts, including elemental atomic number (Z), the Pauling electronegativity (χ), and the coordination number (CN) of the element. (**D**) The visualization of 131 facets with near-optimal ∆E_CO_ identified by ML as potential intermetallic catalysts for CO_2_ reduction. Reproduced with permission from ref [124]. Copyright 2018, Springer Nature

It is well known that structure, particularly atomic arrangements, plays a crucial role in determining catalytic properties. Consequently, compositional and geometric descriptors, which are readily accessible, are intuitively chosen as key indicators of catalytic properties. These descriptors encompass various characteristics, including element type and content, bond lengths, bond angles, atomic radius, coordination numbers, particle geometry (e.g., thickness, dimension, and size), surface area, and pore size. They can be derived from structural characterization techniques or existing databases. As a typical example, Yin et al. used symbolic regression to find elemental and geometric descriptors for the ABO_3_ perovskite structure. The descriptor is defined by μ/t, where μ is the octahedral factor (r_B_/r_O_), and t is the tolerance factor ($\:\frac{{r}_{A}+{r}_{O}}{\sqrt{2}({r}_{B}+{r}_{O})})$. They find that μ/t has a linear correlation with overpotential at a current density of 5 mA cm^−2^ (Fig. 10A). This linear relationship provides insight to tune structure factors, i.e., the radius of A and B sites, to adjust its catalytic performance. A smaller μ and a larger t should lead to higher OER activity. The mechanism behind this can be explained by the radius change caused by the valence states of A and B elements, since increasing valence states will reduce the ionic radii of transition metal. Based on the proposed descriptor and algorithm, they successfully synthesized four new oxide perovskites, Cs_0.4_La_0.6_Mn_0.25_Co_0.75_O_3_, Cs_0.3_La_0.7_NiO_3_, SrNi_0.75_Co_0.25_O_3_, and Sr_0.25_Ba_0.75_NiO_3_ (Fig. 10B), which performance outperformed the current reported Ba_0.5_Sr_0.5_Co_0.8_Fe_0.2_O_3_ perovskite catalyst [123].

In another case study, elemental descriptors were utilized to screen active sites with optimal adsorption energy. Specifically, the researchers investigated an intermetallic system for the electrocatalytic reaction of CO_2_ reduction and H_2_ evolution [124]. Their elemental descriptors included the atomic number of the element (Z), the Pauling electronegativity of the element (χ), and the number of atoms coordinated with the adsorbate (CN) (Fig. 10C). In addition, the absorption energy, ΔE_CO_, were calculated as the output feature. Finally, they identified 131 facets with near-optimal ∆E_CO_, highlighting them as potential intermetallic catalysts for CO_2_ reduction (Fig. 10D). Besides, this work uncovered trends indicating that combinations of elements featuring strong and weak CO adsorption energies are most likely to form catalytically active sites.

Vacancies have long been recognized, both theoretically and experimentally, for their significant role in catalytic processes, making vacancy concentration a valuable predictor of the catalyst activity. As a typical example, the lattice oxygen atoms (or oxygen vacancies) at the perovskite surface have been reported to directly participate in the oxygen electrocatalysis process [125]. Recently, Zhu et al. used the ML algorithm to fit and predict the oxygen vacancy concentration (denoted as δ) of perovskite (ABO_3−δ_) by descriptors including tolerance factor, electronegativity, polarization power, charge, and cation sizes for A-site and B-site [126]. They subsequently demonstrated a volcano-like dependency of ORR activity on oxygen vacancy concentration by experiment. To be specific, the optimal oxygen vacancy concentration (δ) was 0.374 for cobalt-based perovskite materials at 450 °C for high ORR performance. Density function theory (DFT) calculation and analysis revealed the underlying mechanism of this volcano-like dependency: the low oxygen vacancy concentration restricts the lattice oxygen participation in ORR reaction, while the high oxygen vacancy concentration leads to slow oxygen-ion migration with decreased ORR activity. Combining ML prediction with DFT calculation, this work provides a new horizon to guide the rational design of efficient perovskite materials with optimal oxygen vacancy concentration for ORR electrocatalysis.

The development of first-principles-based toolboxes has provided a revolutionary approach to constructing a detailed molecular-level understanding of electrocatalysis. These tools have been instrumental in calculating the adsorption energies of intermediates and examining the electronic structure and stability of catalysts. Such advancements have enabled scientists to elucidate reaction pathways and identify the thermodynamic properties that govern the catalytic activity of specific materials. By leveraging this prior knowledge in descriptor selection, the free adsorption energy (for example, hydrogen adsorption energy, ΔG_H∗_, for HER) is used as figures of merit (output) to evaluate the reaction activity. Simultaneously, intrinsic electronic structures, including work function [127], electron affinity, band gap, the density of states, *d*-band center (*εd*), valence electron number, occupied and unoccupied d states near the Fermi level, and total d electrons, are selected as input descriptors [91].

**Fig. 11:**
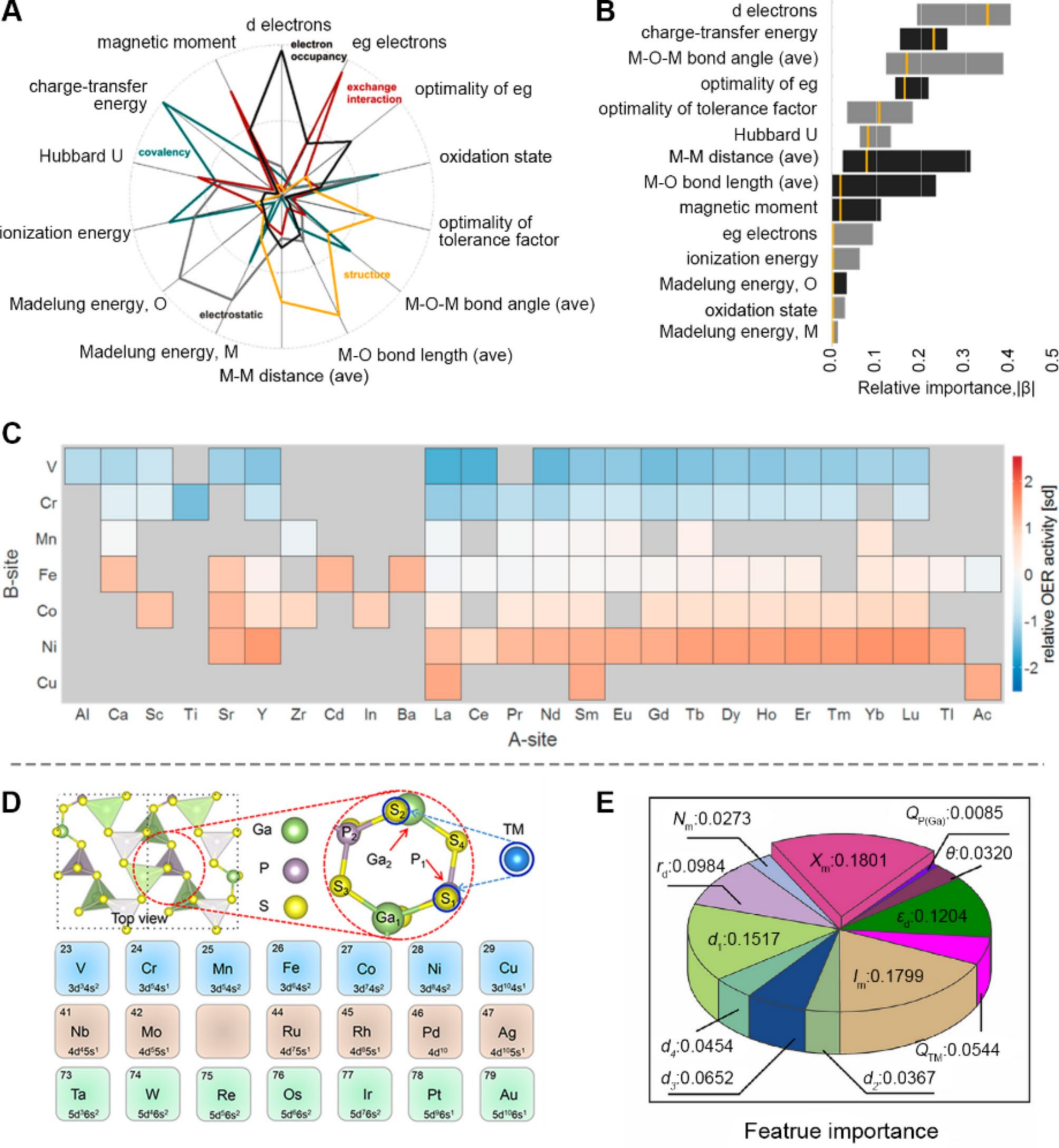
Examples of physiochemical descriptors being key features. (**A**) The classification of 14 descriptors into five descriptor families, including covalency (green), electrostatics (gray), structure (yellow), exchange interaction (red), and electron occupancy (dark gray). (**B**) The relative importance of 14 descriptors analyzed by penalized regression models. (**C**) The predicted relative OER activity for ABO_3_ perovskites. The overall trend shows that the oxides with Fe, Co, Ni, and Cu exhibited higher OER activities than those with V, Cr, and Mn. Reproduced with permission from ref [128]. Copyright 2015, ACS publication. (**D**) The structure diagram of single atom doped 2D GaPS_4_ and the transition metal dopant candidates for S_1_ and S_2_ sites. (E) The feature importance of single atom doped 2D GaPS_4_ materials for HER. Reproduced with permission from ref [129]. Copyright 2023, ELSEVIER publication

However, to date, first-principles calculations and experiments alone have been unable to identify a single or few physicochemical factors that most significantly influence catalytic properties. This is where data mining excels: it can reduce dimensionality to extract an optimal subset of descriptors, thereby highlighting the most influential factors in catalysis. A typical example is the work of Yang et al., who statistically evaluate the descriptors relevant to the OER of ABO_3_ perovskites [128]. First, they performed dimensionality reduction by examining the relationships among 14 general OER descriptors, including the transition-metal redox couple, electrical conductivity, transition-metal d-electron count, transition-metal occupancy, metal-oxygen covalency, and etc. By analyzing the correlation strength between every two descriptors, they reorganized these 14 descriptors into five physical meaning factors, including covalency, electrostatics, structure, exchange interaction, and electron occupancy (Fig. 11A). Next, the penalized regression and factor regression models were selected to assess the importance of these factors. Their results indicated that d-electron count, charge-transfer energy, and M-O-M angle are the top three important factors (Fig. 11B). Furthermore, this statistical learning and data mining tools were applied to predict the relative OER activity for ABO_3_ perovskites, showing that the oxides with Fe, Co, Ni or Cu exhibited higher OER activities compared to those with V, Cr, Mn (Fig. 11C). This trend aligns well with previously reported findings.

Similarly, Liu and coworkers studied the influence of different descriptors in the system of SACs doped 2D GaPS_4_ materials, which possess a large intrinsic band gap that can be adjusted through doping and tensile strain (Fig. 11D), using DFT and ML methods [129]. They found that the electron affinity and first ionization energy are the two most critical descriptors related to the HER behavior, with feature importance of 0.1801 and 0.1799, respectively. In addition to qualitative analysis, graphing and visualization can also help find potential descriptors. For example, Zhao and colleagues explored the potential descriptor for the structure-activity relationship in copper-based alloy nanoparticles by visualizing the optimal adsorption sites for hydrogen on the surface of copper-based alloy nanoparticles [130]. They discovered that the most optimal sites with high HER activity are the bridge sites of the vertex and edge on the shell Cu atoms, so the H adsorption-free energies should be associated with the charge values of two adjacent Cu atoms located on the vertex site and edge site. Hence, they suggest the average charge difference between two adjacent E_Cu_ and V_Cu_ as a descriptor to probe the structure-activity relationship. This finding provides new insights for designing alloy cluster electrocatalysts and exemplifies the process of discovering novel descriptors through data analysis approaches.

#### Data mining for domain knowledge

At the second level, the data-mining process itself becomes a method for adhering to established domain rules, such as Sabatier’s principle. This principle—the golden rule in electrocatalysis—reflects the idea that the adsorption energy of intermediates should be neither too strong nor too weak for optimal catalytic performance, showing non-monotonic dependence. However, many reactions, such as ORR, involve multiple steps and intermediates. As a result, constructing a comprehensive volcano plot becomes impractical without enumerating all possible intermediate-catalyst adsorption energies and corresponding activities. Furthermore, a volcano plot is typically limited to mapping or predicting structure-activity relationships within a specific material family, such as metal single crystals. Data mining provides a viable approach to capture the non-monotonic catalytic activity of families of new materials without the need for extensive first-principles calculations and experimental efforts.

**Fig. 12:**
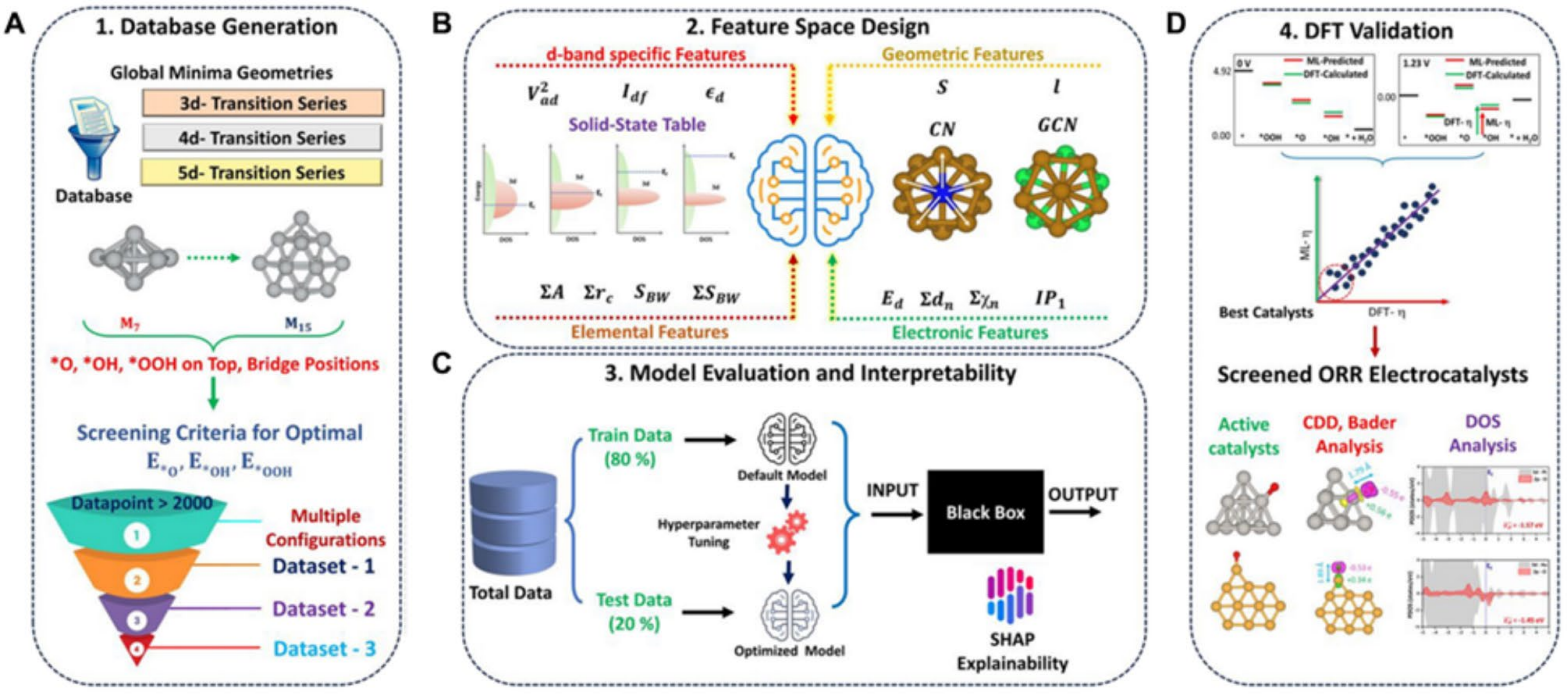
Screening active electrocatalysts using the ML framework. (**A**) Stepwise generation of absorption energy database of *O, *OH, and *OOH on 3d, 4d, and 5d TM subnano clusters. (**B**) Selected descriptors, including d-band specific, geometric, elemental, and electronic features. (**C**) Model training, evaluation, and interpretability. (**D**) DFT validation for predicted electrocatalysts. Reproduced with permission from ref [131]. Copyright 2024, American Chemical Society

For example, Pathak and colleagues employed an ML framework to map the ORR catalytic trend in 3*d* to 5*d* transition metal (TM) subnano clusters [131]. As shown in Fig. 12A, starting from reported optimized geometries of TM subnano clusters with number of atoms in the range of 7 to 15, they adopted a stepwise screening process to filter out the 108 most stable and active configurations with their corresponding adsorption energy of *O, *OH, and *OOH (denoted as E_*O_, E_*OH_, E_*OOH_, respectively) as output. Next, elemental, electronic, geometric, and *d*-band-specific indicators were selected and pre-processed as effective features to define the local environment of the subnano clusters (Fig. 12B). Later, five well-developed ML models available in the Scikit-learn open-source library were employed using the training dataset and evaluated by testing dataset (Fig. 12C). Models with high prediction accuracy were selected for different intermediates, specifically, gradient boosting regression (GBR) for E_*O_ and E_*OOH_, and random forest regression (RFR) for E_*OH_. Furthermore, Shapley additive explanation (SHAP) analysis was conducted to interpret the trained “black box”, essentially extracting the impact of each descriptor on the output. The top three features contain a geometric feature—bond length with a positive effect, and two *d*-band-specific features, including *d*-band filling with a positive correlation and a coupling matrix with a negative correlation, which aligns well with classical *d*-band theory. Finally, the trained models were used to construct the ORR volcano plot for subnanoclusters, simultaneously identifying the top five potential electrocatalysts (Fig. 12D). Notably, the volcano plot displays a rightward shift compared to the bulk metal volcano plot, with Au at the peak instead of Pt, as seen in bulk metals. Such results can be attributed to the size-induced fluxional identity of these clusters, offering deeper insights into breaking the traditional volcano plot.

In another case, multi-descriptor models can enhance the conventional one-dimensional volcano plots derived from the Sabatier principle, transforming them into a multi-dimensional framework. This approach is particularly effective for predicting selectivity in multiproduct reactions. For instance, the competition between CO and H_2_ evolution and the selectivity towards CO or formate products indicate that a simplistic ΔG_CO_^*^ volcano may not sufficiently predict the activity and selectivity of CO_2_RR [132]. Alternatively, to accurately predict the activity and selectivity towards 4 main products of CO_2_RR including formate, CO, C_1+_ (> 2e^−^), and H_2_, a 3D selectivity map is constructed using multiple descriptors, including ∆E_CO_^*^, ∆E_H_^*^, and ∆E_OH_^*^ [133]. These binding energies are predicted by a DFT and structure-free ML model from a previous report [134], which used descriptors of the elemental nearest neighbors to avoid time-consuming DFT calculations. Different regions within this map correlate with preferred products of CO_2_RR, such as the CO formation region with weak binding strength of CO* and OH*, the H_2_ formation region with both strong binding strength with CO* and H*, formate production region with weaker ΔE_CO_* than ΔE_OH_, C_1+_ selective region with moderate ΔE_CO_*, ΔE_OH_*, and ΔE_H_*. In summary, such a thermodynamic framework serves as a valuable tool to classify catalysts into selective regions, enabling the high-throughput discovery of promising CO_2_RR catalysts without exhaustive DFT calculations.

**Fig. 13:**
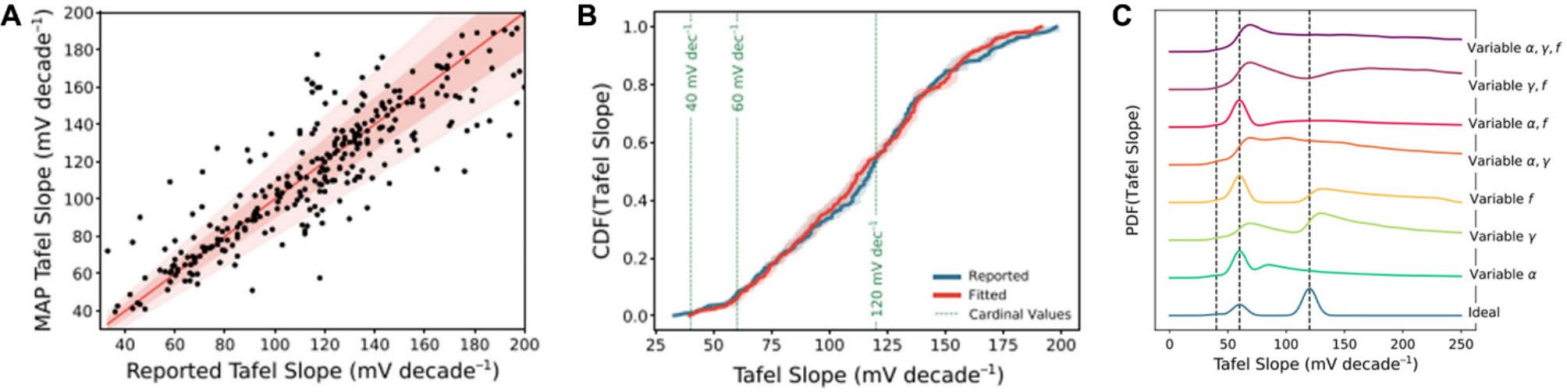
Analysis of cardinal values of the Tafel slope with the Bayesian algorithm. (**A**) The relationship between reported and mean a posteriori (MAP) estimated Tafel slopes. (**B**) Cumulative distribution function of reported Tafel slope (blue) and their refitted result (red). (**C**) The effect of physical nonidealities on kernel density estimates (KDE) of the probability distributions (PDF) over the Tafel slope. Reproduced with permission from ref [135]. Copyright 2021, Springer Nature

Moreover, the statistical nature of data mining renders it a good approach to examine some empirical or idealized rules. Taking the Tafel slope (Eq. 1) as a typical example, theoretically, several “cardinal values” (such as 40, 60, 120 mV/decade) exist when the total number of electrons transferred in elementary steps before or in the rate-determining step (RDS) is 0 or 1. Therefore, these “cardinal values” are used as metrics to evaluate the kinetics of reactions occurring on certain catalyst surfaces. To reevaluate this rule, Manthiram et al. developed a Bayesian data analysis approach to statistically re-analyze Tafel slopes from reported CO_2_ reduction literature without human bias [135]. As shown in Fig. 13A-B, the experimental obtained Tafel slope does not show a preference for these cardinal values. Additionally, they explored the underlying reasons by randomly introducing physical nonidealities into the model, such as fluctuations of electrochemical double layer (*α*), formation of surface diploe (*γ*), and protrusion of an electrode-adsorbed species (*f*). Figure 13C illustrates that these three physical nonidealities can indeed cause significant changes in the distribution of Tafel slopes, providing a possible explanation for the observed behavior.

1 $$\:T\equiv\:{\left.\frac{d\eta\:\:}{d{\text{l}\text{o}\text{g}}_{10}{i}_{\text{k}\text{i}\text{n}}}\:\right|}_{\left|\eta\:\right|\gg\:{k}_{B}T/e}$$

One electrocatalysis area has not been fully explored is that, although electric field is the driving force that mediates reactions, its effects at the microscopic level (i.e. chemical bonds, molecules, single atoms) are not well understood due to the difficulty in quantitative measurements. Thanks to the rapid advancements of in-situ spectroscopy and ultrafast spectroscopy in recent years, Luo et al. proposed that the spectra can be used to correlate the effect of electric fields on electrocatalysis and further quantify this response through ML models [136]. Leveraging the artificial intelligence model, the infrared and Raman spectra calculated by DFT revealed that the asymmetric stretching vibrations of the CO_2_ molecule in Raman spectra were closely related to the CO_2_ adsorption free energy on metal-doped graphitic C_3_N_4_ (g-C_3_N_4_ ) under electric field. Detailed findings indicate that as the electric field intensity increases, the CO_2_ adsorption energy strengthens on catalysts, and charge transfer also becomes more pronounced. This result contributes to the development of domain knowledge, confirming the most relevant vibrational modes related to adsorption free energy and revealing the microscopic level influence of electric field on catalyst’s surface. Though the spectra data used in this work is calculated by DFT, this work provides a future direction towards the integration of in-situ spectra techniques with the data mining strategies.

#### Domain knowledge for interpretable models

**Fig. 14:**
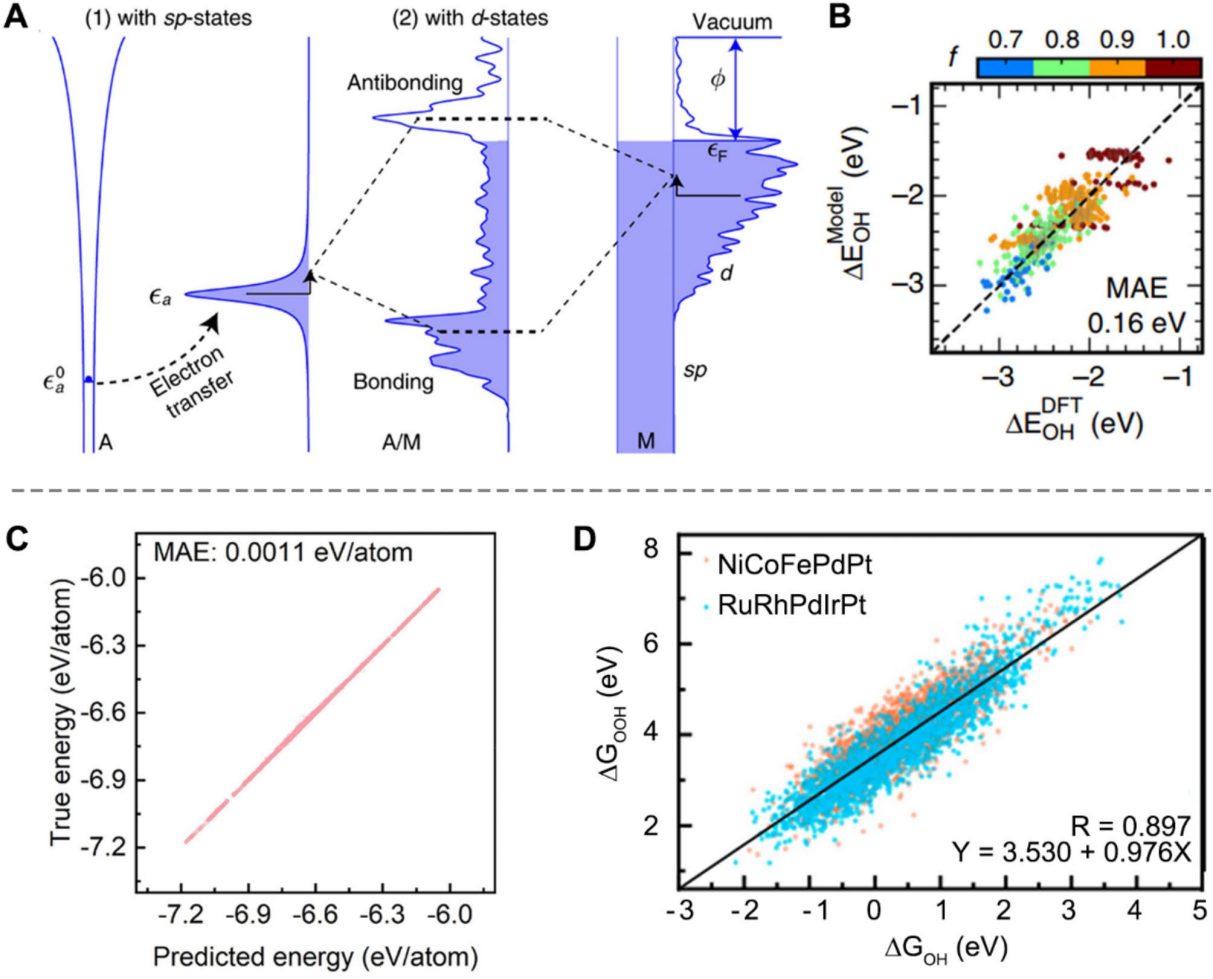
Domain knowledge from first-principles based calculations for interpretable models. (**A**) Schematic representation of chemical bonding on TM surfaces in *d*-band theory. (**B**) The plot of DFT-calculated adsorption energies as a function of model-predicted adsorption energies of *OH at the {111}-terminated intermetallic atop sites. Reproduced with permission from ref [137]. Copyright 2020, Springer Nature. (**C**) True versus predicted energy of Ni-Co-Fe-Pd-Pt. (**D**) Relationship between ΔG_OH_ and ΔG_OOH_ on NiCoFePdPt and RuRhPdIrPt. Reproduced with permission from ref [138]. Copyright 2023, Elsevier

At the third level, prior knowledge or rules derived from first-principles calculations in limited chemical spaces are used to train interpretable models. These models potentially surrogate costly DFT calculations, enabling the efficient prediction of targeted properties and facilitating high-throughput catalyst screening. For example, Xin et al. developed a Bayesian inference ML approach by learning *d*-band theory from a single crystal dataset, allowing it to predict the bonding strength of reaction intermediates on various intermetallic and alloy surfaces [137]. In this method, DFT calculations were conducted on {111}-terminated metal surfaces to obtain parameters related to *d*-band theory (including energy contribution from the *sp*-band, adsorbate resonance energy relative to the Fermi level, *sp*-band chemisorption function, orbital overlap coefficient, and orbital coupling coefficient (Fig. 14A). These parameters were then used to train a Bayesian statistical learning model, achieving a mean-absolute error (MAE) of approximately 0.1–0.2 eV for intermediate adsorption on more complex metal sites (Fig. 14B).

In another attempt, an atomic graph attention (AGAT) network was developed to predict the surface potential energy landscape of high entropy electrocatalysts, where graphs are used as input rather than feature sets [138]. This graph-based input structure enables AGAT to explicitly capture the interactions between atoms in a material, which is particularly advantageous for high-entropy materials where such interactions can be complex and varied. An adsorption energy spectra database was built for equiatomic and non-equiatomic RuRhPdIrPt and NiCoFePdPt through DFT calculations to train the AGAT model. The trained model demonstrates high prediction accuracy, achieving an MAE of only 1.1 meV/atom for energy on the test set (Fig. 14C). Notably, this model is inherently interpretable, as its attention scores explicitly reflect the message passing between nodes, providing insights into the underlying atomic interactions. Later, they found that predicted adsorption Gibbs energies of intermediates on high entropy alloy for ORR statistically follows a linear scaling relationship (Fig. 14D), which aligns well with the case for pure metal.

**Fig. 15:**
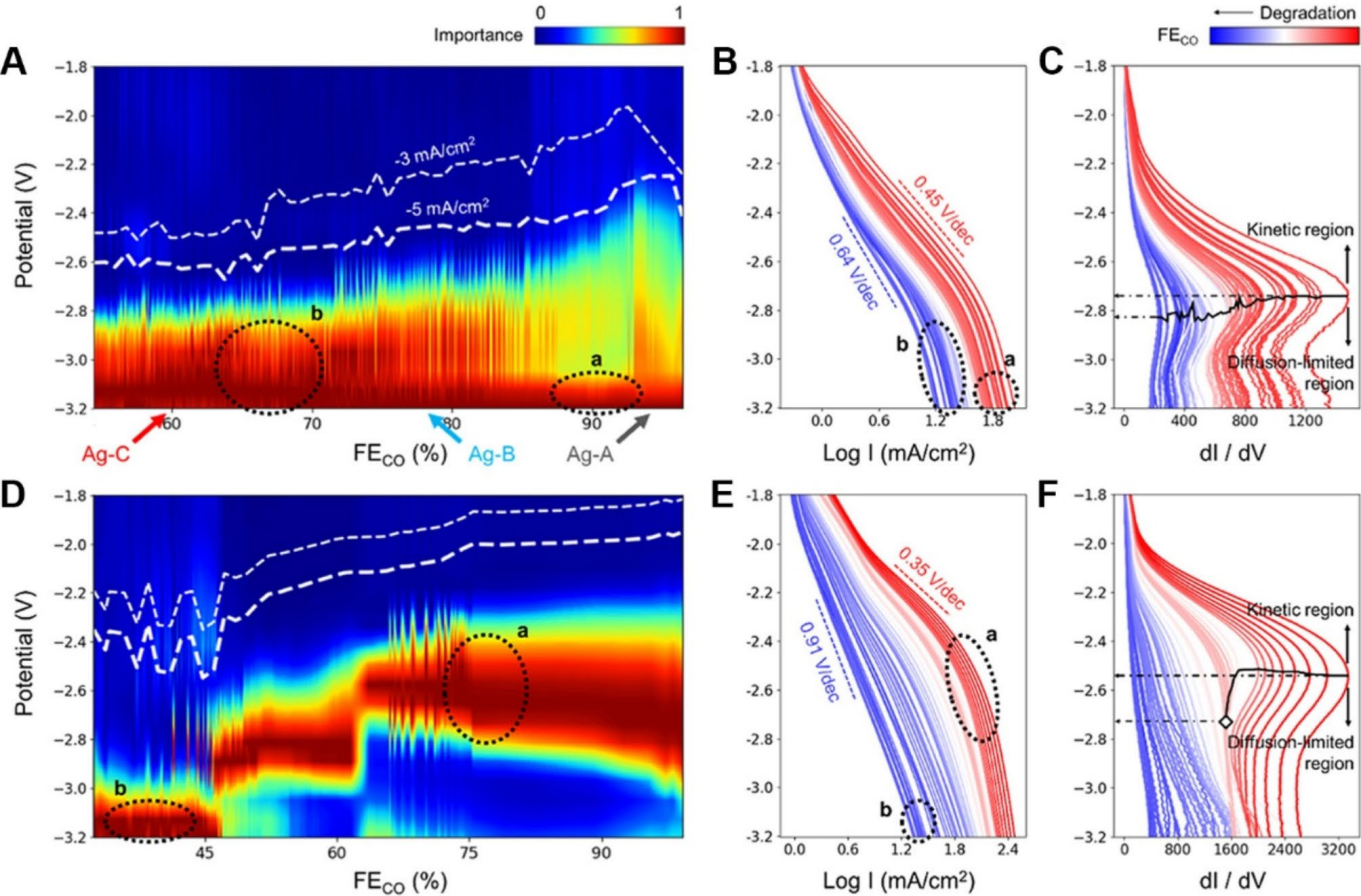
Integrating domain knowledge and explainable artificial intelligence for interpretable models. (**A**) Expanded Grad-CAM attention map for Ag electrocatalysts. (**B**-**C**) Tafel plots and dLSV plots of randomly selected LSV curves. Specific transition points between kinetic and diffusion-limited regions are represented as a black line on dLSV plots. (**D**-**F**) Expanded Grad-CAM attention map, Tafel plots, and dLSV plots for Ni – N/C. Reproduced with permission from ref [139]. Copyright 2025, American Chemical Society

The LSV measurements are widely performed to obtain metrics for evaluating electrocatalysts, such as onset potential, overpotential, exchange current density, and Tafel slope plot. Among them, the Tafel plot is composed of a reaction kinetics-controlled region and a mass transfer-controlled region. Recently, by leveraging domain knowledge and explainable artificial intelligence (XAI), Lee et al. interpreted the origin of electrocatalysts degradation [139]. Specifically, they applied gradient-weighted class activation mapping (Grad-CAM), an XAI algorithm, to visualize the key voltage regions of LSV curves (Fig. 15A, D) for Ag and nickel single atom anchored on nitrogen-doped carbon (Ni-N/C) catalysts. For Ag catalysts, the highlighted attention region that plays a decisive role in predicting catalytic performance corresponds to the diffusion-limited region in the attention map (Fig. 15A-C), thus they demonstrated that the extrinsic mass transport was the governing factor for Ag catalyst degradation. While for Ni-N/C, the highlighted attention areas focus on both the intrinsic kinetic region and extrinsic diffusion limited region (Fig. 15D-F), indicating the intrinsic kinetic exhibits significant attention in Ni − N/C degradation. Further, experimental characterization confirmed that the loss of active sites in Ni − N/C after catalyst degradation. In summary, the infusion of domain knowledge into XAI supported ML approach enabled the interpretation of the black-box ML model result. This work not only identifies key degradation mechanisms in electrocatalysts but also demonstrates the potential of interpretable ML in the rational design and development of advanced electrocatalysts.

#### Integration of in-situ technique with data mining

**Fig. 16:**
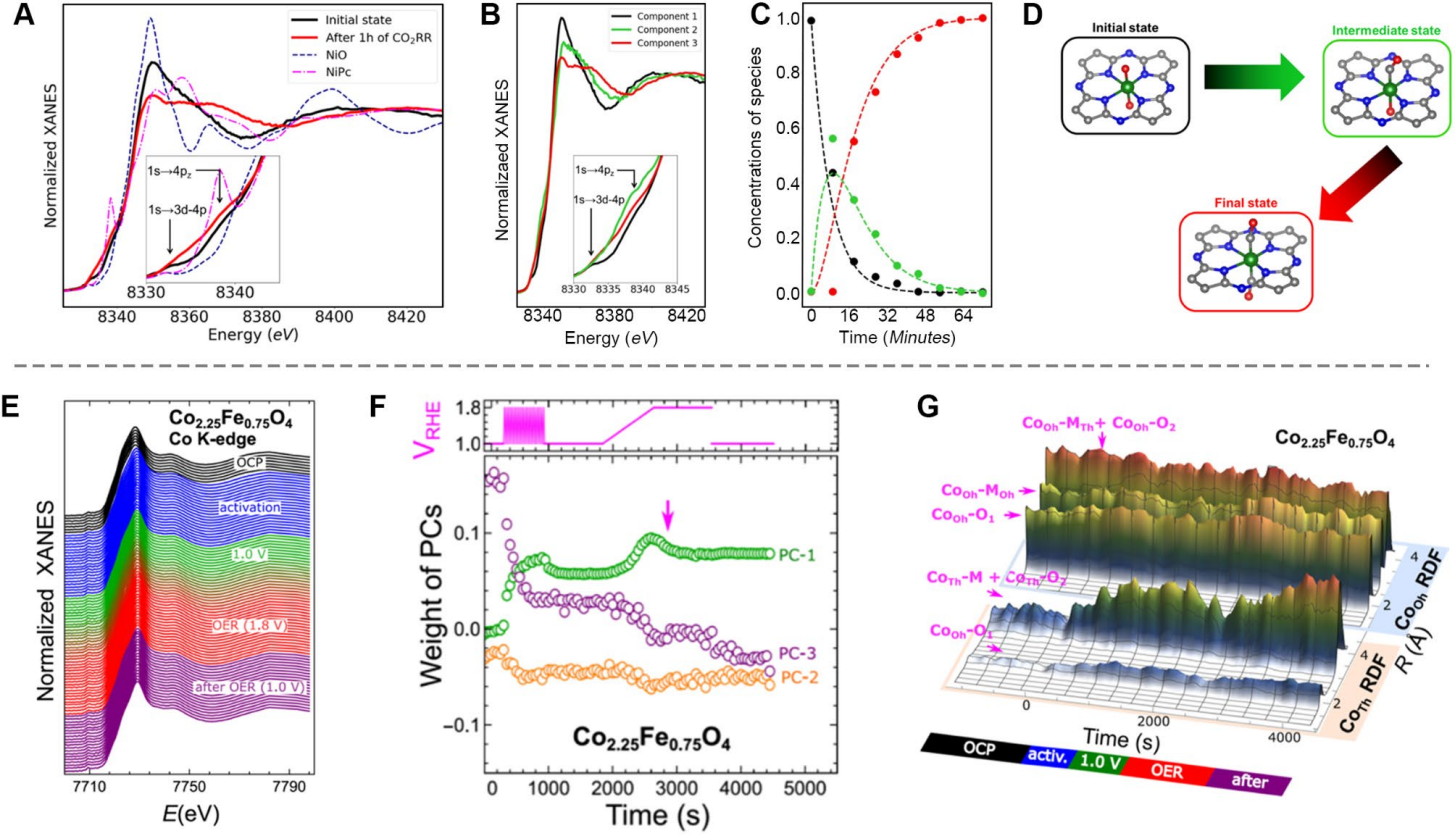
Integration of in-situ experiments with data mining. (**A**) Operando Ni K-edge XANES spectra of the heat-treated Ni TMNC catalyst collected before and after the CO_2_RR. (**B-C**) Extracted XANES spectra (**B**) and corresponding concentration profiles (**C**). (**D**) The proposed reaction mechanism based on the PCA analysis and EXAFS fitting. Reproduced with permission, from ref [143]. Copyright 2023, American Chemical Society. (**E**) Operando Co K-edge XANES for the Co_2.25_Fe_0.75_O_4_ catalyst under OER conditions. (F) Evolution of the weights of the PC-1, PC-2, and PC-3 for the Co_2.25_Fe_0.75_O_4_ under operando condition. (G) Evolution of RDFs for tetrahedrally and octahedrally coordinated Co sites in Co_2.25_Fe_0.75_O_4_ under operando condition. Reproduced with permission, Reproduced with permission, from ref [141]. Copyright 2023, American Chemical Society

In-situ and operando experiments typically generate large and complex data streams, including spectra, images, and time-resolved profiles [140]. To effectively analyze these datasets, prior knowledge, such as standardized data for model systems, is essential. However, this information is often lacking, particularly for short-lived intermediates. Consequently, analyzing these complex datasets to extract valuable insights into catalytic behavior can be quite challenging. To tackle this, advanced data mining methods—such as principal component analysis (PCA) [141], NN algorithms [142], multivariate curve resolution − alternating least square (MCR-ALS) analysis [142], and non-iterative reconstruction—can be employed to extract meaningful insights and enhance our overall understanding of the experimental results. Taking a powerful in-situ/operando technique, XAS, as an example, we present how ML or data mining techniques are leveraged in XAS data analysis to assist mechanism extraction during the electrocatalysis process. For instance, Martini et al. studied the reaction mechanism of Ni-based transition metal-nitrogen-doped carbons (TMNC) catalyst in CO_2_RR by combining XAS and advanced data analysis [143]. Figure 16A shows Ni K-edge XANES spectra of Ni TMNC before and after the reaction, with well-matched absorption edge position but remarkably distinct XANES features compared to that of available reference Ni^2+^ materials, suggesting unique local structure motif surrounding Ni single atom in TMNC. Therefore, standard linear combination fitting cannot be applied for spectral decomposition. Alternatively, they employed unsupervised ML methods, i.e., PCA, to identify the principal components (PCs) resulting in the variation of time-dependent XANES spectra. Three PCs were identified, suggesting that three kinds of Ni species—initial state, intermediate state, and final state—are present in Ni TMNC during CO_2_RR. Next, the transformation matrix approach was applied to transform the identified three abstract PCs into actual XANES spectra (Fig. 16B, C). Subsequently, they used a supervised ML-based XANES fitting algorithm to establish a non-linear correlation between local structure parameters and Ni K-edge XANES profiles, with structural parameter sets determined by an Adaptive Sampling approach and XANES spectra calculated by FDMNES (Finite Difference Method Near Edge Structure) as training dataset. The model was then used to deduce the corresponding atomic structures of these three Ni species, which were validated by Reverse Monte Carlo simulation. Last but not least, they proposed the mechanism scheme (Fig. 16D), showing CO adsorbates gradually replacing O or OH axial ligands on Ni atoms during CO_2_RR.

The effectiveness of the advanced data mining approach has also been demonstrated in understanding the in-situ/operando data related to the structural and chemical evolution of catalysts. In an operando study of spinel-like Co_x_Fe_3−x_O_4_ OER catalysts using quick X-ray absorption fine structure spectroscopy (QXAFS), Timoshenko et al. employed the PCA method to extract the primary PCs causing the variation in the obtained QXAFS dataset during OER (Fig. 16E) [141]. Three PCs (PC-1, PC-2, and PC-3) were identified, correlating with the Co oxidation state, the fraction of Co species, and the Co − O distance, respectively. Thus, the evolution of Co species can be deduced from the trends in the weight of these PCs. For example, the PC-1 plot indicates that the oxidation state of Co species increases as the potential ramps from 1.1 V to 1.8 V, followed by a decrease when resting at 1.8 V, suggesting partial dissolution of the material (Fig. 16F).

Furthermore, to gain insight into structural transformations, a NN based EXAFS analysis method (NN-EXAFS) was employed to map the relationship between EXAFS features and the partial radial distribution functions (RDFs). Compared to conventional EXAFS analysis methods, the trained NN-EXAFS model is more adept at handling EXAFS spectra with asymmetric shapes that cannot be accurately fitted using traditional methods. Figure 16G shows typical analysis results from NN-EXAFS applied to Co_2.25_Fe_0.75_O_4_, revealing distinct evolution of RDF for the tetrahedrally coordinated Co (Co_Th_) within a spinel-like motif and octahedrally coordinated Co (Co_oh_) within a rocksalt-like structure under activation, working, and post-reaction conditions. It is observed that the activation process leads to the irreversible transformation of the rocksalt-like structures into a spinel-like motif for the Co sites, as evidenced by the growing concentration of Co_Th_ and the decrease in the Co_Oh_−O bond. Lastly, they attributed the edge-sharing Co^3+^−O_6_ octahedral units, formed through the local structural transformations of Co sites, serve as active sites for the OER.

## Conclusion and outlooks

In summary, this article highlights the advancement of guided catalyst design through in-situ experimental techniques and data-mining approaches. On the one hand, the catalytic mechanism is in-situ probed using developed experimental methods, such as SECM, SECCM, TIRF-SR, SMFM, XAS, XPS, Raman, IR, and DEMS, among others. The insights gained from these processes significantly enhance our understanding of critical catalytic processes, including reactant adsorption, charge transfer, formation of intermediate, and production of reaction products, thereby informing and guiding further catalyst development.

On the other hand, due to its intrinsic ability to handle large datasets, data mining has been rapidly applied to catalyst screening, serving as a complementary tool to HTE. Data mining excels in managing the high-dimensional search spaces provided by HTE and other databases, particularly in identifying key descriptors that govern targeted catalytic properties and highlight associated trends. Besides, while HTE is invaluable, it is impractical to exhaustively screen the whole chemical space due to constraints such as time, cost, and labor intensity. In contrast, data mining approaches leverage identified trends and analyzed results to efficiently explore vast chemical spaces, serving as a powerful approach to accelerating catalyst discovery. Families such as single-atom doped materials, 2D materials, metal-organic frameworks, and alloys are commonly explored using these approaches in catalysis research.

Despite progress and advancements, several challenges remain to be addressed to effectively implement in-situ techniques and data mining in rational electrocatalyst design.

(1) **Integration of various in-situ techniques.** Fully unveiling catalytic processes using a single in-situ experimental technique is challenging. For example, while in-situ EC-STM can pinpoint the localization of active sites with atomic resolution, it cannot track the formation of reaction intermediates. This constraint poses significant challenges in deciphering the complex reaction pathways characteristic of multi-electron-transfer catalytic reactions. A common approach to address this challenge is the integration of different in-situ experimental techniques. For example, combining SMF microscopy and X-ray spectroscopy can bridge the gap between high-resolution spatial imaging and electronic structure analysis. Moreover, integrating in-situ experiments with data mining can yield new insights into catalytic mechanisms. Data mining techniques, such as Bayesian and clustering algorithms, can analyze large datasets generated by in-situ techniques (e.g., XAS, XPS, or IR spectroscopy) to identify correlations between reaction conditions and catalytic activity. By processing spectral data with advanced computational models, data mining can potentially highlight information related to transient intermediates that are difficult to isolate experimentally.

(2) **High spatiotemporal techniques.** The current in-situ experimental techniques struggle to simultaneously achieve high spatial resolution (at the nanometer to angstrom scale) and high temporal resolution (at the picosecond to femtosecond scale). This limitation poses challenges in fully capturing the spatial distribution of active sites and short-lived transient intermediates during catalytic processes. To this end, it is imperative to develop or integrate techniques that can bridge this gap. Emerging approaches offer promising directions, such as combining ultrafast spectroscopy with advanced electron microscopy or other spectroscopic methods.

(3) **Stability evaluation with data mining**. Data-mining-driven catalyst design approaches primarily rely on optimizing catalytic performance based on activity criteria. However, a significant step in transitioning catalysts from laboratory research to industrial application is ensuring robust catalytic stability under harsh industrial operating conditions. Industrial processes often subject catalysts to extreme thermal, mechanical, and chemical stresses, requiring them to maintain performance over extended periods. For example, the U.S. Department of Energy has set a durability target requiring proton exchange membrane water-splitting devices to operate continuously for 80,000 h under cycling conditions, with an average degradation rate of 2.0 mV/kh [144]. Consequently, developing data mining approaches prioritising stability-evaluation criteria is essential, particularly under demanding conditions. This includes analyzing large datasets to identify patterns and factors influencing catalyst deactivation, such as sintering, poisoning, and structural degradation. Advanced ML algorithms can integrate experimental data from accelerated stability tests and operando monitoring to predict long-term performance trends and identify failure mechanisms.

(4) **The quality and dimension of the database.** The distribution and dimensionality of the database are crucial aspects of the effectiveness of a data-mining approach. A well-distributed, multi-dimensional, high-quality dataset forms the foundation for successful data mining. The database should include complementary descriptors to enhance the model’s robustness and flexibility. For instance, both experimental and theoretical descriptors related to the figure of merits should coexist. This is important because calculated descriptors are often under ideal conditions, whereas those obtained from actual experiments can vary based on numerous factors, specific test conditions (pH, electrolyte concentration, electrode activation), and even catalysts with the same chemical formula, different morphologies, specific surface areas, and micro-nano structures [94].

(5) **High-throughput experimental platforms.** High-throughput platforms are highly sought after to expedite the development of electrocatalysts. However, such platforms are relatively scarce in practice. There is an urgent need for guidelines and protocols to establish integrated high-throughput platforms that facilitate closed-loop electrocatalyst preparation, performance testing, and structural characterization [145].

(6) **Domain knowledge infusing in the data mining process.** Researchers in the field of catalysis should consider leveraging the powerful capabilities of data mining not only for catalyst screening and optimization but also as a key component of their daily research activities. Despite some initial efforts to integrate domain-specific knowledge and rules into data mining approaches, this practice remains in its early stages. Advancing this integration could significantly enhance the interpretability and effectiveness of data mining in uncovering new insights, guiding hypothesis generation, and establishing sophisticated models that reflect complex catalytic phenomena.

(7) **Applying data mining approaches to analyze in-situ/operando data**. (i) Leveraging prior knowledge to evaluate fitted results. For example, the effective application of NN algorithms to EXAFS analysis requires extensive domain knowledge, including a chemical understanding of EXAFS spectra (e.g., atomic coordination environment, oxidation state, and structural deformation), as well as key machine learning principles such as model architecture selection, strategies to avoid overfitting, and methods for incorporating physical constraints into the modeling process. (ii) Consistency in data preprocessing. In-situ/operando experimental datasets may exhibit signal shifts due to environmental conditions, as well as missing or ambiguous values, outliers, and noise. To ensure the reliability of analysis, it is crucial to maintain consistency in data preprocessing. Standardized key steps for data cleaning, including zero-point correction calibration, background subtraction, interpolation, and normalization are required. (iii) Cross-validation with multiple algorithms and characterizations. It is important to employ various algorithms to assess in-situ/operando data, as each algorithm comes with its unique advantages and limitations. By comparing results obtained through different approaches, researchers can evaluate the accuracy and robustness of the findings. Furthermore, we encourage the use of in-situ/operando data derived from different techniques that capture the same process, as this complementary information can enhance the reliability of the analysis and provide a more comprehensive understanding of the underlying phenomena.

## Data Availability

Not applicable.

## Abbreviations

A-Lab: Autonomous laboratory for inorganic powder synthesis
AFM: Atomic force microscopy
AGAT: Atomic graph attention
AP-XPS: Ambient-pressure XPS
CFP: Carbon fiber paper
CO_2_ RR: CO_2_ reduction
DEMS: Differential electrochemical mass spectrometry
DFT: Density functional theory
dLSV: Derivative of LSV
DT: Decision trees
EC-SHINERS: Electrochemical shell-isolated nanoparticle-enhanced Raman spectroscopy
EC-STM: Electrochemical scanning tunneling microscopy
EELS: Electron energy loss spectrum
EXAFS: Extended X-ray absorption fine structure
FDMNES: Finite difference method near edge structure
FE: Faraday efficiency
g-C_3_N_4_: Graphite C_3_N_4_
GBR: Gradient boosting regression
GIXRD: Grazing incidence X-ray diffraction
GPR: Gaussian process regression
Grad-CAM: Gradient-weighted class activation mapping
HAADF STEM: High-angle annular dark-field scanning transmission electron microscopy
HAB: Hexaaminobenzene
HEA: High-entropy alloy
HER: Hydrogen evolution reactions
HOPG: Highly oriented pyrolytic graphite
HRTEM: High-resolution TEM
HTE: High-throughput experiments
ICP-MS: Inductively coupled plasma mass spectrometry
IR: Infrared
ITO: Indium tin oxide
KDE: Kernel density estimates
LDH: Layered double hydroxide
LDOS: Local density of states
MAE: Mean-absolute error
MAP: Mean a posteriori
ML: Machine learning
NN: Neural networks
NN-EXAFS: Neural-network based extended X-ray absorption fine structure analysis method
OCP: Open-circuit potential
OCV: Open-circuit voltage
OER: Oxygen evolution reaction
ORGANA: Robotic assistant for automated chemistry experimentation and characterization
ORR: Oxygen reduction reaction
PDF: Probability distributions
PPy-CuPcTs: Copper phthalocyanine-3, 4’, 4’’, 4’’’-tetrasulfonic acid tetrasodium salt doped polypyrrole film
QXAFS: Quick X-ray absorption fine structure spectroscopy
RDF: Radial distribution functions
RDS: Rate-determining step
RFR: Random forest regression
RHE: Reversible hydrogen electrode
SAC: Single-atom catalysts
SECCM: Scanning electrochemical cell microscopy
SECM: Scanning electrochemical microscopy
SEM: Scanning electron microscope
SERS: Surface-enhanced Raman spectroscopy
SG/TC: Substrate generation/tip collection
SHAP: Shapley additive explanation
SHINERS: Shell-isolated nanoparticle-enhanced Raman spectroscopy
SI-SECM: Surface-interrogation SECM
SMFM: Single-molecule fluorescence microscopy
SOAP: Smooth overlap of atomic positions
SRIR: Synchrotron radiation infrared
TEM: Transmission electron microscopy
TM: Transition metal
TOF: Turnover frequency
UV-Vis: Ultraviolet-visible
XAI: Explainable artificial intelligence
XANES: X-ray absorption near edge structure
XAS: X-ray absorption spectroscopy
XPS: X-ray photoelectron spectroscopy
